# Immunomodulatory topographies regulate myofibroblast differentiation and influence fibrous encapsulation of glaucoma drainage devices

**DOI:** 10.1016/j.bioactmat.2026.01.012

**Published:** 2026-02-02

**Authors:** Phani Krishna Sudarsanam, Ralph J.S. van Mechelen, Tim J.M. Kuijpers, Christian J.F. Bertens, Els Alsema, Juul Verbakel, Nick R.M. Beijer, Henny J.M. Beckers, Jan de Boer

**Affiliations:** a Department of Biomedical Engineering, Institute for Complex Molecular Systems, Eindhoven University of Technology, Eindhoven, 5600, MB, the Netherlands; b University Eye Clinic Maastricht, Maastricht University Medical Center+, Maastricht, 6202, AZ, the Netherlands; c Centre for Health Protection, National Institute for Public Health and the Environment, Bilthoven, 3720, BA, the Netherlands

**Keywords:** Macrophage, Fibroblast, Foreign body response, Glaucoma, Surface micro topography

## Abstract

Elevated intraocular pressure (IOP) is the primary driver of glaucoma, and lowering IOP remains essential for preventing vision loss. Glaucoma drainage devices (GDDs) help reduce IOP but often fail due to fibrosis. This study identifies surface micro topographies capable of modulating the fibrotic response to GDDs both *in vitro* and *in vivo*. Using the TopoChip high-throughput platform (2176 topographies), we fabricated poly(styrene-block-isobutylene-block-styrene) SIBS based micro topographies and screened them for their effects on human Tenon fibroblast adhesion and transdifferentiation, as well as primary macrophage attachment and cytokine expression. These screens revealed micro topographies that differentially regulate fibroblast and macrophage behaviour, enabling the selection of three candidate designs for *in vivo* evaluation. When incorporated into glaucoma shunts implanted in rabbits, these micro topographies produced distinct tissue responses compared with smooth controls, including differences in collagen deposition and density at the implant interface. Overall, this work demonstrates that engineered micro topographies can modulate wound healing around GDDs and provides a foundation for design-driven strategies to improve implant performance. Future studies will focus on long-term implantation to optimize therapeutic outcomes.

## Highlights

• Foreign body reaction is one of the primary challenges faced by the implants after glaucoma surgery.
• Micro topographies play a crucial role in modulation of biomaterial mediated cell responses.
• This research presents a novel approach to identify micro topographies crucial to counter foreign body response.
• High-throughput screening enables discovery of new biomaterials that modulate fibroblast and macrophage phenotypes.

## Graphical abstract

**Figure:**
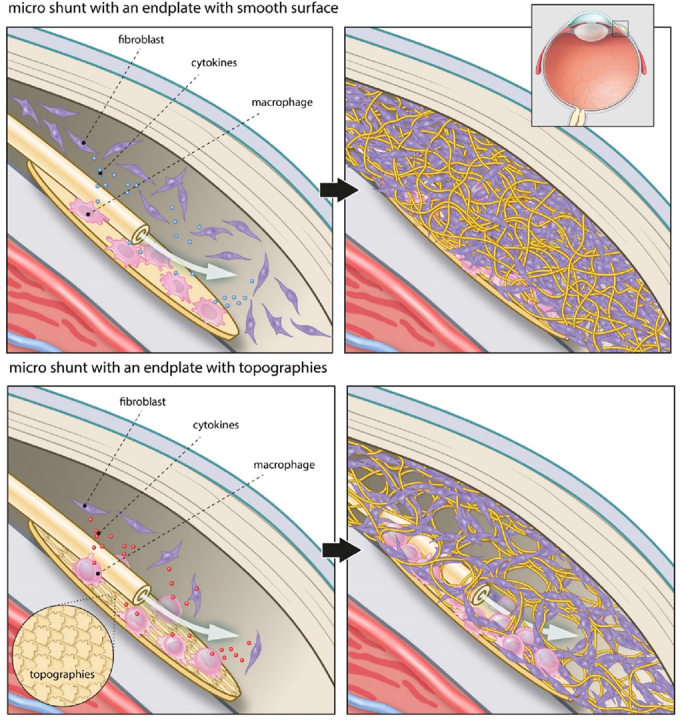

## 1 Introduction

Glaucoma, a degenerative disease of the optic nerve, is the leading cause of irreversible blindness, affecting over 70 million people worldwide in 2020 [1]. Despite its global prevalence, the etiology and pathophysiology of glaucoma remain incompletely understood. Elevated intraocular pressure (IOP), however, is recognized as the primary risk factor and lowering IOP remains the only proven therapeutic strategy capable of slowing or halting vision loss in most patients. Increased IOP results from heightened resistance within the aqueous humor (AqH) outflow system. Initial treatments include IOP-lowering medications and laser procedures, but when these options are insufficient, surgical intervention with glaucoma drainage devices (GDDs) becomes necessary [2]. These devices facilitate the drainage of AqH from the anterior chamber into the subconjunctival/sub-Tenon's space, forming a small reservoir or "bleb". However, a major challenge with GDDs is the exacerbated wound healing response, leading to excessive fibrosis and ultimate implant failure. AqH flows through a tube connected to an endplate positioned in the subconjunctival space, but an excessive foreign body response (FBR) can cause the endplate to be encapsulated by dense fibrotic tissue, obstructing AqH outflow and resulting in elevated IOP.

FBR is a multi-stage immune reaction to implanted materials such as stents, catheters, and prosthetic devices [[3], [4], [5]]. Different polymers used in these implants evoke distinct FBR responses. At the final stage of FBR, fibrous tissue encapsulation often results in device failure. For instance, brain implants fail due to insulating fibrous layers [6], breast implants may require removal due to severe encapsulation [7], and GDDs experience failure due to fibrosis blocking the filtration bleb [8,9]. After implantation, tissue damage and protein adsorption trigger rapid acute inflammation, with neutrophils arriving first, followed by monocytes that become macrophages. These macrophages initially adopt a pro-inflammatory state, releasing cytokines that amplify inflammation [10,11]. With non-degradable materials, chronic inflammation develops, driving fibroblast recruitment and extracellular matrix (ECM) deposition [12]. As the response progresses, macrophages shift to anti-inflammatory, pro-fibrotic phenotypes that stimulate fibroblast-to-myofibroblast differentiation through factors like TGF-β1. The resulting collagen-rich fibrotic capsule can impair or fail implanted devices [13].

A wealth of literature demonstrates that monocytes and macrophages are sensitive to material properties and change their adhesive and inflammatory phenotype accordingly, and importantly also their secretory profile [14,15]. While previous studies have extensively investigated macrophage mediated FBR *in vivo*, the critical role of fibroblast activation often remains underexplored. Notably, fibroblasts can independently drive fibrosis through an autocrine loop, where TGF-β1 stimulates fibroblasts to secrete additional TGF-β1, further reinforcing the fibrotic response. This fibroblast driven process is particularly relevant in glaucoma surgery, as human Tenon fibroblasts (HTFs) interact with AqH derived growth factors, contributing to bleb failure [16].

The severity of the FBR is also influenced by biomaterial properties such as shape, surface chemistry, stiffness, and topography [17,18]. Studies indicate that surface roughness affects FBR, as seen in textured breast implants, where biofunctionalization with IL-4 reduces pathological fibrosis compared to smooth implants [19]. Similarly, glaucoma implants with textured surfaces, such as the Ahmed implant, exhibit higher tenon fibroblast adhesion, correlating with an increased incidence of failure due to fibrotic encapsulation compared to Molteno and Baerveldt implants. These findings highlight the significant role of topography in cell adhesion and fibrosis modulation after implantation [20]. These observations warrant an in depth understanding of the role of micro topography in cell responses specifically with cell types that are known to affect the FBR. Our research group previously performed screens using mesenchymal stromal cells with a library of different micro topographies which were able to influence cell proliferation as well as create unique cytokine secretion profiles [21,22].

To address this challenge, our study explores topographical modifications in glaucoma implants to control fibrosis and enhance implant longevity. Existing GDDs, including silicone and polypropylene based designs, present limitations in bleb survival and implant success rates due to their material properties and design specifications [23]. However, the PERSERFLO® MicroShunt, a SIBS polymer-based device, has demonstrated low failure rates [24]. In this manuscript, we used the elastomer SIBS which was developed in the recent past as a material inducing a very low FBR. SIBS has gained prominence as a low FBR polymer, used in drug eluting stents and next generation glaucoma devices [25,26]. Despite its low fouling properties [27], SIBS implants still fail due to excessive fibrosis at the AqH outflow site, resulting in bleb failure. This could be correlated to the activation of fibroblasts through aqueous humor via mechanotransduction pathways that can affect cell differentiation [28]. Growth factors such as MCP-1, TGF-β2, and VEGF in AqH, coupled with mechanical mismatch between implants and tissues, further amplify the immune response and fibrosis [[29], [30], [31]].

Various studies have explored diverse strategies for fabricating topographical features on biomaterial surfaces. Techniques such as lithography, deposition, etching, and advanced 3D writing methods have provided precise tools to create micro- and nano-scale topographies [32]. These engineered surface features can be tailored to influence cellular behavior and are increasingly utilized in a wide range of biological and biomedical applications, including tissue engineering, regenerative medicine, and implantable devices. Nano topographical features on biomaterials guide cell behavior by influencing cytoskeletal organization, integrin signaling, and protein adsorption from the extracellular matrix. Understanding these interactions is key to designing next-generation implants that enhance tissue integration while addressing challenges like bacterial colonization and sterilization [33]. However, very few studies have examined the role of surface topography at the implant tissue interface in GDDs. One study showed that hydrophilic coatings and micropatterned endplates on Ahmed implants altered capsule thickness in rabbit models, suggesting that surface modifications influence FBR [34]. Another study demonstrated that grooved surface designs, combined with cyclosporine coatings, enhanced bleb survival and reduced fibrotic tissue formation in implanted rabbits. These findings underscore the potential of surface topography modulation in mitigating fibrosis in GDDs [35]. In this study, we investigate whether topographical modifications can improve the patency of GDDs by controlling fibrosis at the outflow site. Our approach focuses on modifying the endplate surface with bioactive micro topographies to regulate the fibrotic response. Two primary research questions guide this investigation:

Can FBR be tailored through surface micro topographies that modulate macrophage and fibroblast responses at the implant site?

Can the induced tissue responses from distinct micro topographies prevent fibrotic bleb failure and enhance shunt function?

To address these challenges, we propose a novel glaucoma shunt inspired by the PRESERFLO® MicroShunt design [36], in which surface topography is engineered to regulate fibrotic tissue formation at the bleb site. We hypothesize that the biomaterial interface influences fibrotic progression through secreted factors that ultimately affect long-term implant survival. To systematically investigate topography-driven cellular responses, we employed the TopoChip high-throughput screening platform, which contains 2176 unique micro topographies [37]. Building on previous work demonstrating the influence of surface topography on mesenchymal stem cell proliferation and macrophage phenotype [38,39], we performed three complementary TopoChip screens: (1) assessment of human Tenon's fibroblast (HTF) transdifferentiation using α-smooth muscle actin (α-SMA) as a marker, (2) quantification of HTF attachment across diverse topographies, and (3) characterization of macrophage phenotype by profiling pro- and anti-inflammatory cytokine secretion in response to different surfaces. Through this combined approach, our goal is to develop a novel bioactive GDD capable of modulating the wound healing response and improving bleb longevity after surgery.

## 2 Results

### 2.1 Fabrication of SIBS TopoChips and bioassay development

**Fig. 1:**
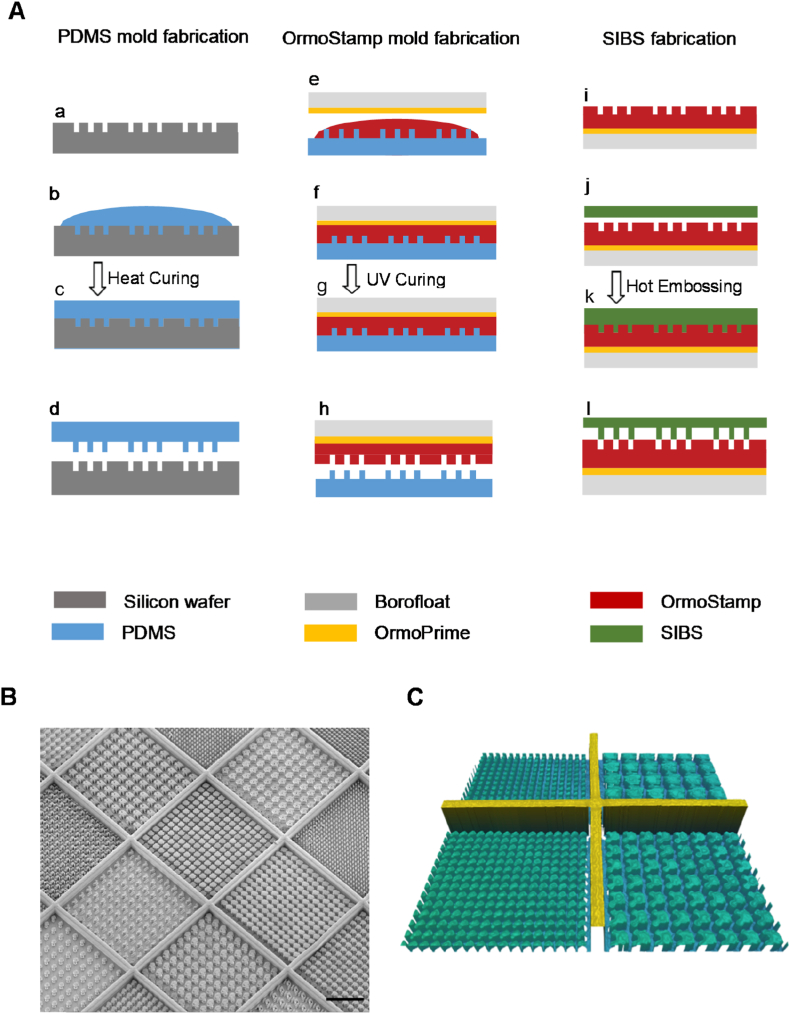
**SIBS TopoChip fabrication.** (A) Step by step fabrication process to produce topographical imprints with different topographies. (B) SEM image representing a section of a TopoChip fabricated with SIBS polymer showing the replication of features from the mold onto the polymer (Scale bar 100 μm). (C) Profilometric analysis of height shows the replication of topographies with walls separating each TopoUnit.

We fabricated TopoChips with SIBS polymer due to their biocompatibility, following a previously described process [38] (Fig. 1A). Scanning electron microscopy (SEM) confirmed the fidelity of micro topographies (Fig. 1B). The height of the features was measured by interferometry, and we confirmed that the micro topographies have a height of 10 μm in each TopoUnit (Fig. 1C). Bright field microscopy ensured no damage post hot embossing.

Before cell culture experiments, the SIBS TopoChips were plasma oxygen treated (see Materials and Methods) to counter the hydrophobic nature of SIBS polymer. Without treatment, we were unable to keep the TopoChips submerged in a culture medium in a 6-well plate. We retrospectively tested differences in macrophage attachment with and without plasma treatment in a special device to submerge untreated SIBS surface and observed no differences in the design of the micro topographies between the top and bottom hits from our TopoChip screen (Fig. S1).

**Fig. 2:**
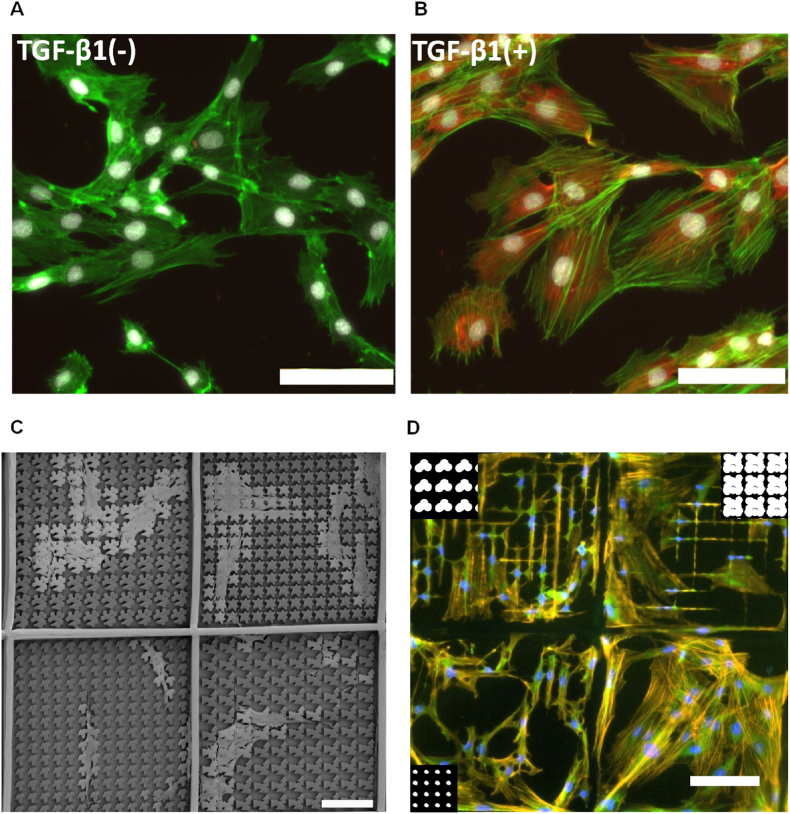
**Bioassay for human tenon fibroblasts (HTFs) trans differentiation.** HTFs seeded on SIBS polymer were exposed to 10 ng/ml TGF-β1. (A) Control without TGF-β1. Cells show typical spindle shaped structures. (B) High expression of α-SMA (in red) with pronounced stress fibres is seen when HTFs are exposed to TGF-β1 (green: F-actin, white: nuclei, red: α-SMA; scale bar 100 μm). (C) SEM image of cells on a SIBS TopoChip showing distinct morphological differences based on the topographical design (scale bar: 100 μm). (D) Fluorescent image of a TopoChip seeded with HTFs and treated with TGF-β1. Flat surface on the bottom right surrounded by three different Topo units (T-1 on left, T-34 on top left, T-33 on top right, binary images of the topographies places inlay) each showing differences in cell organisations and formation of stress fibres (orange: F-actin, green: α-SMA, blue: nuclei; scale bar 100 μm).

For HTF screening, we established a bioassay to evaluate fibroblast transdifferentiation into myofibroblasts by measuring α-SMA expression in response to TGF-β1, a known inducer of HTF transdifferentiation. Exposure to TGF-β1 led to a notable increase in α-SMA expression (Fig. 2A and B), which we quantified using a custom-built CellProfiler pipeline (data not shown). Additionally, TGF-β1 promoted stress fiber maturation (Fig. 2B). We employed the TopoChip due to its diverse topographical patterns, which influence cell attachment and phenotype. SEM images taken 48 h post seeding revealed distinct morphological changes based on topography design (Fig. 2C). These structural differences affected cytoskeletal organization and cell shape. Flat surfaces encouraged a spread-out morphology, whereas cells on structured micro topographies appeared either constrained between pillars or formed a carpet like layer on top. These cellular responses correlated with topography size and spacing (Fig. 2D).

### 2.2 Identification of micro topographies inducing myofibroblast differentiation

**Fig. 3:**
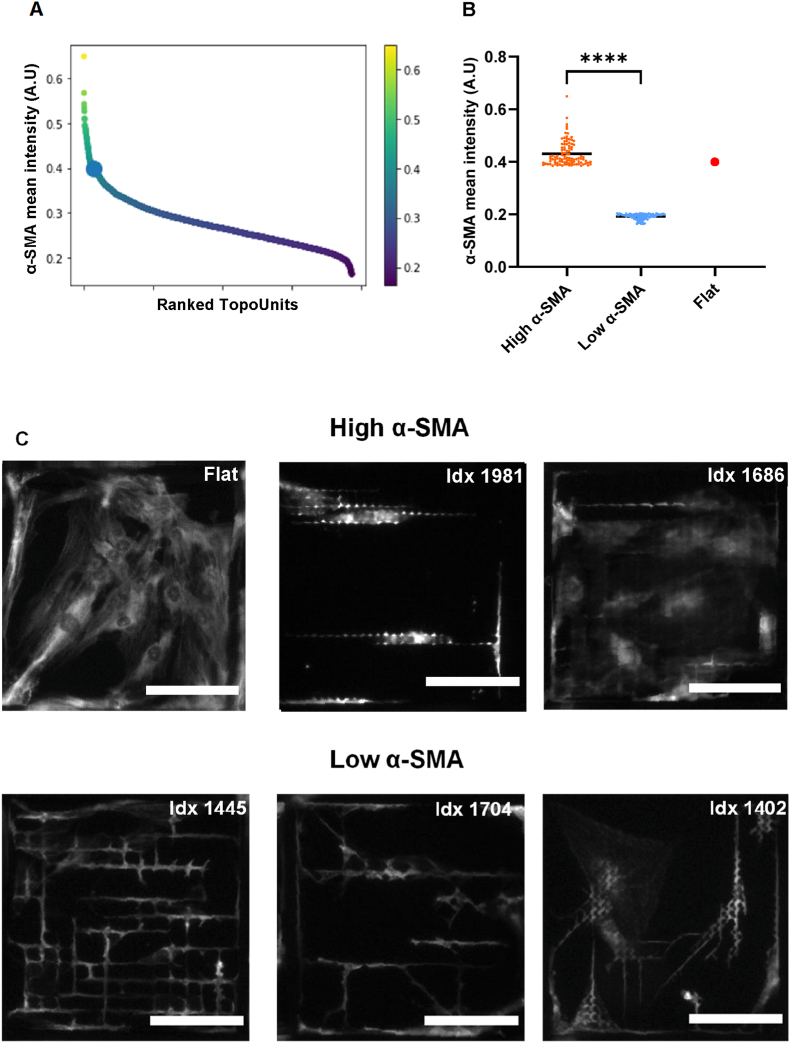
**TopoChip screening on SIBS polymer to assess α-SMA expression.** (A) Ranked α-SMA expression across individual topographies, presented as mean α-SMA fluorescence intensity normalized per cell. The flat surface control is indicated by the bold dot. (B) Quantification of mean α-SMA intensity for 100 high- and low- α-SMA expressing topographies, demonstrating a significant difference between the two groups; the flat surface is shown in red (n = 10, ∗∗∗∗P < 0.0001). (C) Representative immunofluorescence images of α-SMA staining illustrating differences in cell morphology and actin organization between high α-SMA topographies (Idx 1981, 1686, Flat) and low α-SMA topographies (Idx 1445, 1704, 1402) (Scale bar: 100 μm).

To identify micro topographies that promote a favorable tissue response in glaucoma devices, we conducted an HTF TopoChip screen. Each SIBS TopoChip contained four flat surface replicates and duplicate topographies, with 20 replicates per design across ten chips. HTFs were cultured for 48 h with 10 ng/ml TGF-β1, mimicking draining bleb conditions. The screen aimed to identify micro topographies that suppress α-SMA expression relative to the flat surface. Cells were stained for α-SMA, F-actin, and DNA, and analyzed using a custom image analysis pipeline. Notably, the flat surface was among the highest α-SMA expressing surfaces, with nearly 75 % of micro topographies displaying lower α-SMA expression (Fig. 3A). A significant two-fold difference in α-SMA levels was observed between the top 100 highest and lowest α-SMA-expressing micro topographies (Fig. 3B).

**Fig. 4:**
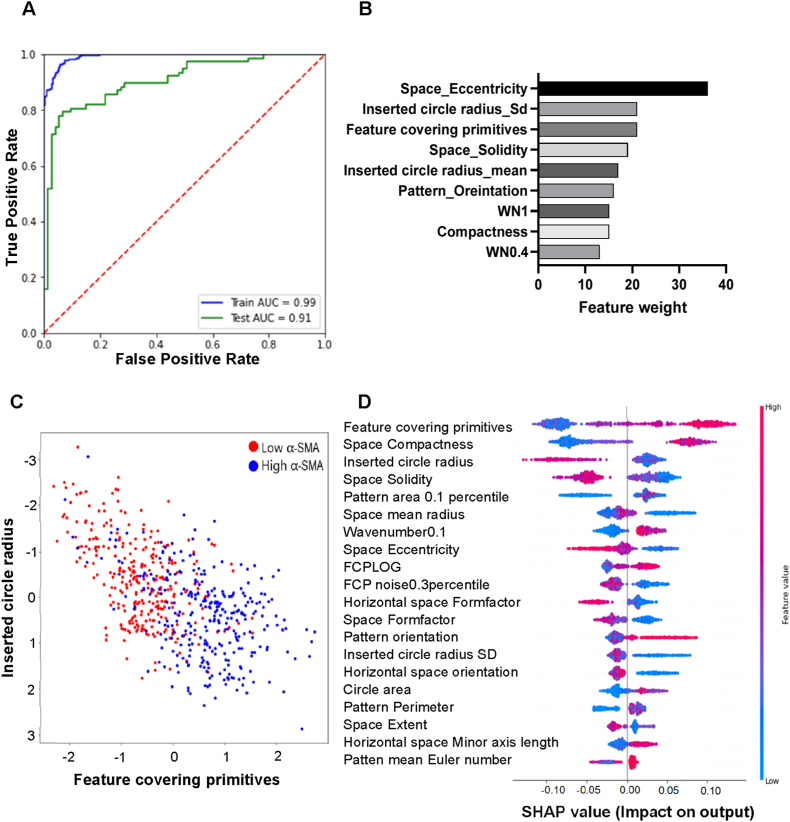
**Correlation between topographical design descriptors and α-SMA expression.** (A) Receiver operating characteristic curve with area under the curve (AUC) demonstrating the predictive performance of the extreme gradient boosting (XGB) model. (B) Plot showing the relative weights of TDDs in the trained machine learning model; eccentricity of pillar spacing and inserted circle radius emerged as the dominant descriptors distinguishing high and low α-SMA expressing topographies. (C) Scatter plot illustrating the distribution of two representative TDDs feature covering primitives and inserted circle radius for the 300 highest (blue) and 300 lowest (red) α-SMA expressing topographies. (D) Beeswarm plot of SHAP values highlighting the contribution and directionality of selected TDDs used in the predictive model.

Morphological analysis revealed that high α-SMA expressing cells were well spread (e.g., flat surface, T-1686) or strongly confined between pillars (T-1981). Conversely, low α-SMA cells were mostly moderately confined (T-1445, T-1704) or restricted in spreading (T-1402) (Fig. 3C). To correlate α-SMA expression with topographical design descriptors (TDDs), we applied an eXtreme Gradient Boosting (XGB) model, using the top 300 highest and lowest α-SMA expressing topographies. The model achieved 91 % accuracy in classifying high vs. low α-SMA surfaces, validating that similar designs produce similar cell responses (Fig. 4A). Key TDDs influencing α-SMA suppression included pillar spacing eccentricity, inserted circle radius, and feature covering primitives (Fig. 4B). A clear separation is observed between high and low α-SMA expressing topographies driven by two TDDs: feature covering primitives and inserted circle radius, which respectively represent feature size and pillar spacing and are key determinants in the design of the micro topographies (Fig. 4C). Lower feature covering primitives and compact pillar spacing correlated with reduced α-SMA expression, as shown by the Beeswarm SHAP analysis (Fig. 4D). Overall, larger pillars with small spacing promoted higher α-SMA expression, while moderate spacing and smaller pillars allowed cell spreading, reducing α-SMA levels (Fig. S2).

### 2.3 Micro topographies induce differential cell attachment of HTFs

**Fig. 5:**
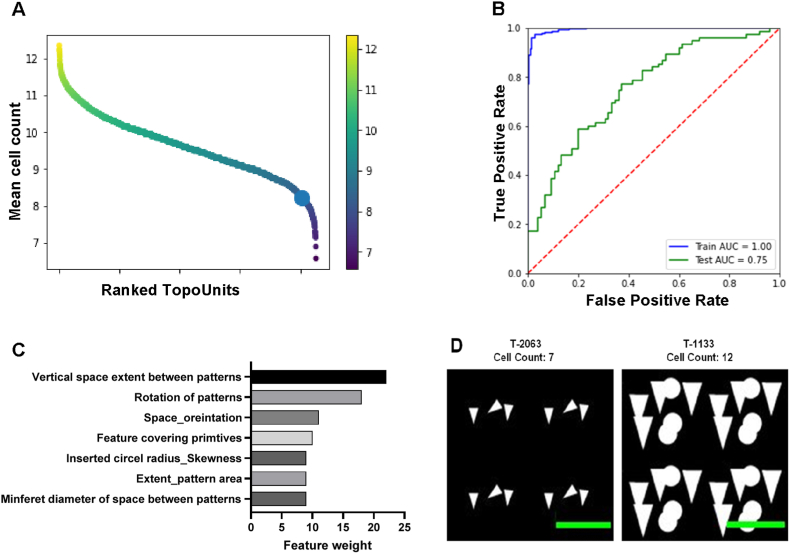
**TopoChip screening to quantify differential HTF attachment.** (A) Ranked distribution of HTF cell number across individual topographies with the flat surface indicated by the bold dot. (B) Area under the curve (AUC) plot shows the predictive power of the model generated with the XGB algorithm. (C) Feature importance analysis showing the relative contribution of TDDs influencing HTF cell number; vertical spacing and rotational parameters of the patterns were strongly associated with cell attachment. (D) Representative design images of topographies T-2063 (low cell count) and T-1133 (high cell count) illustrating differences in feature size and feature spacing (Scale bar: 20 μm).

To evaluate HTF attachment, a second TopoChip screen was conducted. HTFs were seeded in complete medium, subjected to 24-h serum starvation, and then cultured on ten SIBS TopoChips in complete medium. After 48 h, cells were fixed and DNA stained to assess attachment, while serum starvation was included to examine cell cycle activation. Using custom CellProfiler pipeline, micro topographies were ranked by HTF cell density, revealing that 75 % of topographies supported higher attachment than the flat surface (Fig. 5A). An XGB machine learning model identified key topographical design descriptors (TDDs) predicting cell attachment with 75 % accuracy (Fig. 5B). Vertical pattern spacing and pillar orientation were the most influential TDDs (Fig. 5C). Higher attachment correlated with larger pattern areas and narrower spacing, while smaller features with wider spacing led to lower cell adherence (Fig. 5D). HTFs favored densely covered surfaces over those with intermediate spacing, whereas surfaces with small features and large spacing had reduced cell adherence.

### 2.4 Control of macrophage attachment by topographical design

**Fig. 6:**
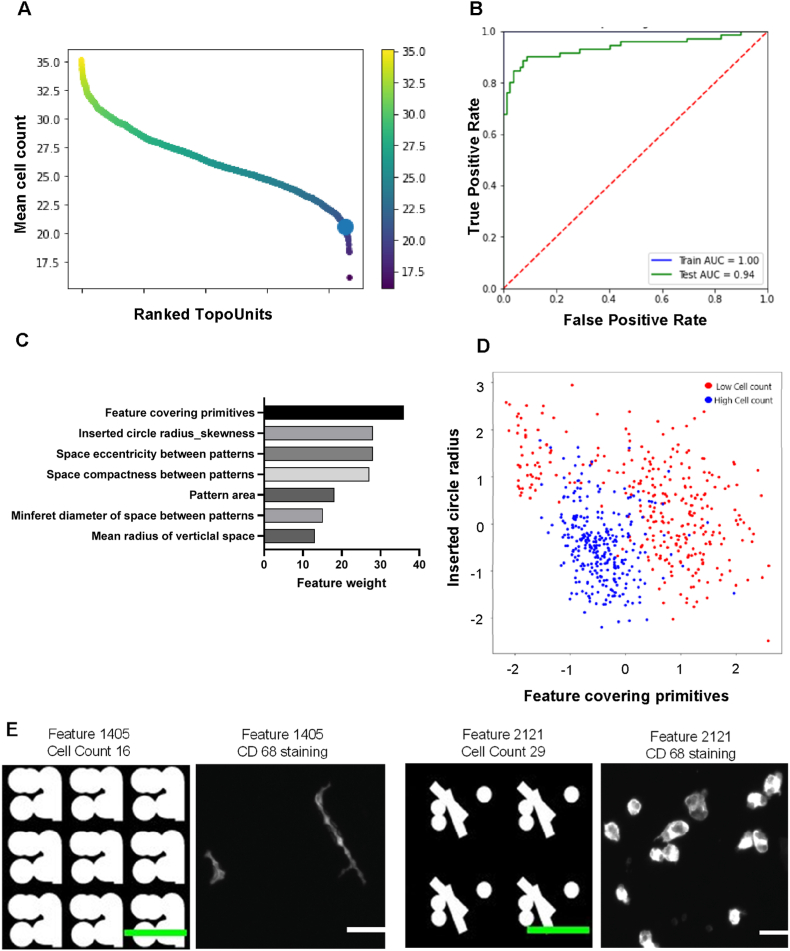
**Macrophage screening on SIBS TopoChips.** (A) Ranked distribution of macrophage cell numbers across individual TopoChip topographies, with the flat surface control indicated by the bold dot; the flat surface exhibited lower cell numbers than approximately 90 % of the tested topographies. (B) Area under curve (AUC) plot showing the predictive value of the model generated by the XGB algorithm. (C) Feature importance analysis showing the relative contribution of TDDs in the macrophage attachment model; feature covering pattern and inserted circle radius skewness showed strong influence on macrophage cell number. (D) Scatter plot illustrating the distribution of two representative TDDs that distinguish topographies inducing high and low macrophage cell count. (E) Representative design and corresponding immunofluorescence images of a low cell count topography (T-1405) and a high cell count topography (T-2121) (Scale bar: design image: 20 μm; Fluorescent image: 100 μm).

In the third SIBS TopoChip screen, we assessed macrophage binding, a critical step in the foreign body response. Our goal was to identify high and low binding surfaces and determine TDDs correlating with macrophage attachment. Monocytes were seeded onto ten SIBS TopoChips with GM-CSF to induce macrophage differentiation. After ten days, cells were fixed and stained for CD68 and DNA, confirming CD68-positive macrophage differentiation (data not shown). Image analysis quantified attached macrophages, ranging from 17 to 35 cells per TopoUnit, with the flat surface at the lower end (Fig. 6A). An XGB machine learning model achieved 94 % accuracy in predicting macrophage binding based on TDDs (Fig. 6B). Key TDDs included feature covering primitives (feature size), inserted circle radius skewness (variation in pillar spacing), and eccentricity and compactness (confinement between features) (Fig. 6C). A scatter plot illustrates clear clustering of high and low binding micro topographies based on these TDDs (Fig. 6D). Low binding surfaces featured large areas between pillars, limiting macrophage adhesion. In contrast, high binding surfaces had pillars of ∼5–10 μm, where macrophages preferentially adhered (Fig. 6E). This behaviour aligns with macrophage attachment patterns observed in other polymeric topographies [39].

### 2.5 Selection of micro topographies for *in vivo* glaucoma shunt model

Following three TopoChip screens, we identified three distinct micro topographies for further *in vivo* testing in a rabbit glaucoma shunt model. Given that macrophages and fibroblasts drive the FBR, we selected micro topographies based on their ability to influence macrophage binding, fibroblast attachment, and myofibroblast differentiation and categorized them as follows:

1. *Quiet Encapsulation* – Low macrophage binding with high HTF attachment
2. *Pro Encapsulation* – High macrophage binding and elevated α-SMA expression
3. *Anti-Fouling* – Minimal attachment of both macrophages and HTFs

**Fig. 7:**
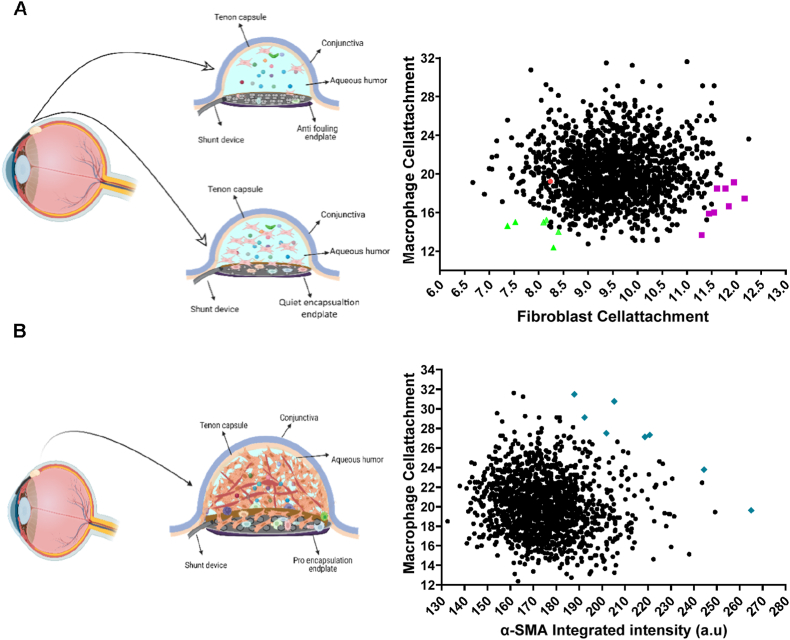
**Selection of three classes of topographies from Topochip screens.** (A) Scatter plot of fibroblast and macrophage attachment used to identify topographies that promote *anti-fouling* or *quiet encapsulation* responses. Schematic illustrations depict the expected tissue outcomes for each condition. Topographies selected for validation of *anti-fouling* responses are highlighted in green, while those associated with *quiet encapsulation* are shown in magenta. (B) Scatter plot used to identify *pro encapsulation* topographies based on macrophage attachment and integrated α-SMA intensity. The accompanying schematic illustrates the expected *in vivo* response. Selected *pro encapsulation* topographies are highlighted in blue.

Scatter plots (Fig. 7A and B) revealed that while most micro topographies support intermediate cell binding, distinct subpopulations do exist, confirming that macrophages and HTFs interact differently with surface topographies. This underscores the value of screening-based approaches in identifying surfaces with complex biological effects. From these findings, 32 candidate micro topographies were selected (colored dots in Fig. 7), replicated on larger surfaces, and validated using the original screening protocols to validate the cell responses observed from the TopoChip screenings. Based on these results, T-509 was identified as the optimal *anti-fouling* topography (Fig. S3).

### 2.6 Cytokine expression profiles as a selection criterion for pro- and quiet encapsulation topographies

**Fig. 8:**
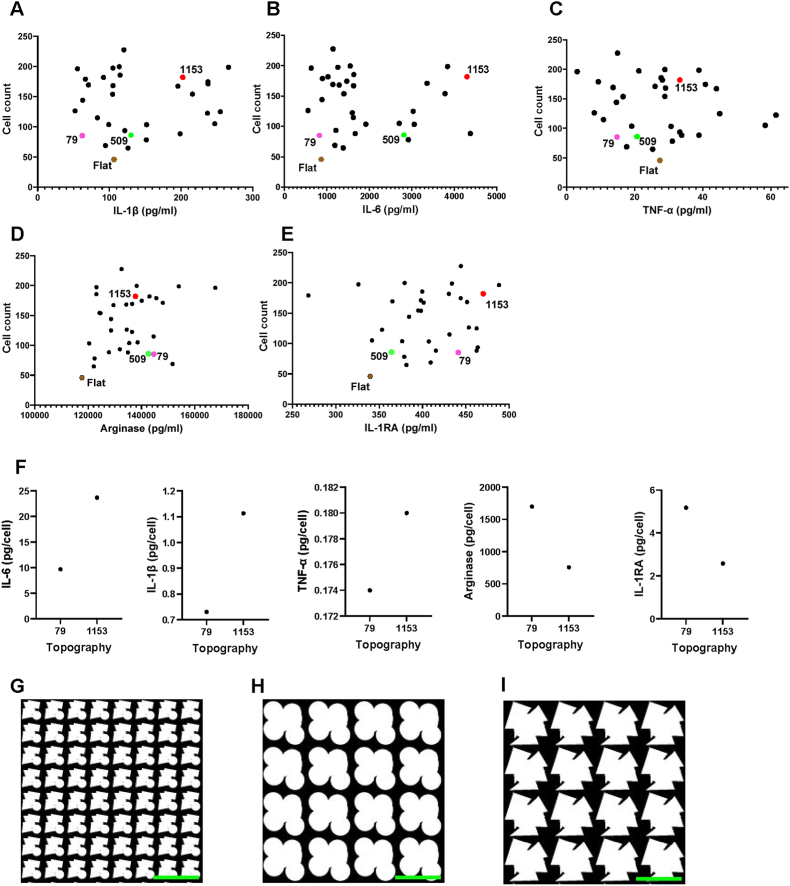
**Multiplex cytokine analysis of topography-mediated cytokine secretion**. (A–E) Scatter plots showing correlations between cytokine expression and cell number across 32 topographies selected from the TopoChip screens. The three topographies chosen for subsequent *in vivo* evaluation are highlighted: (*T-79: Quiet encapsulation; T-509: Anti-fouling; T-1153: Pro encapsulation*). (F) Normalized cytokine levels for selected pro- and anti-inflammatory markers for T-79 and T-509 following normalization to cell number. The multiplex assay was performed on day 3 (*n* = 1). (G–I) Representational binary images of micro topographies selected for the *in vivo* experiments T-79. T-509, T-1153 respectively (Scale bar: 20 μm).

**Fig. 9:**
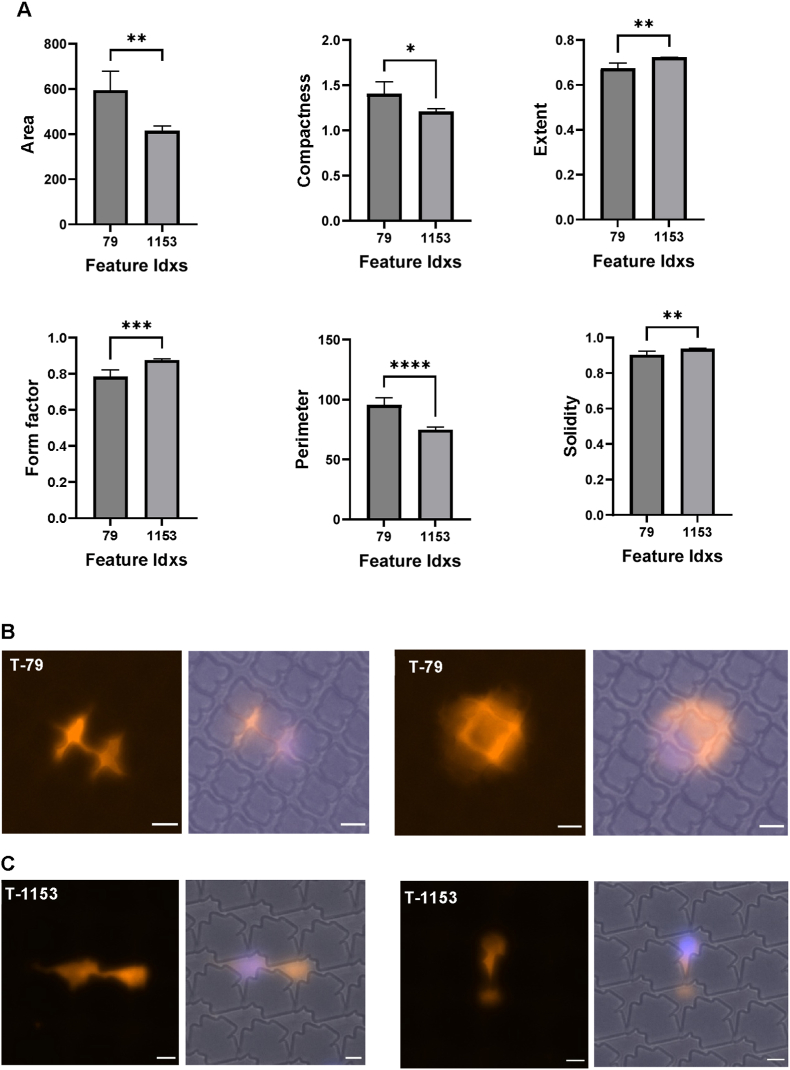
**Macrophage morphological fingerprint in response to micro topographies. (**A)Quantification of selected macrophage morphological parameters for cells cultured on topographies T-79 and T-1153 (n = 2). (B) Representative immunofluorescence images with bright-field overlay of macrophages on T-79, showing confined attachment or increased cell spreading with coverage over individual features.(C) Representative immunofluorescence images with bright-field overlay of macrophages on T-1153, demonstrating a more elongated morphology localized between closely spaced features.(Orange: cell membrane; blue: nuclei; Scale bar: 50 μm).

To further classify *pro-* and *quiet encapsulation* topographies, we analyzed the cytokine expression profiles of macrophages cultured on different topographies. Monocytes were seeded, stimulated with GM-CSF, and conditioned media were collected on days 3 and 6. We assessed cytokines linked to pro inflammatory (IL-1β, IL-6, TNF-α) and anti-inflammatory (IL-1RA, arginase) responses. Cytokine levels varied significantly across topographies. IL-6 levels, for example, ranged from 600 ng/ml on low scoring topographies to 4400 ng/ml on high scoring topographies. Notably, T-79 (*quiet encapsulation*) exhibited low pro-inflammatory and high anti-inflammatory cytokine levels, while T-1153 (*pro encapsulation*) showed the opposite trend (Fig. 8A–E). Thus, T-79 was selected for its low macrophage binding, high HTF attachment, and anti-inflammatory profile, whereas T-1153 was chosen for its high macrophage binding, high α-SMA expression, and pro inflammatory profile. Additionally, macrophage morphology differed between the two topographies. Cells on T-79 were more spread out with larger area and perimeter, while those on T-1153 appeared elongated with higher form factor, extent, and solidity (Fig. 9A). Membrane staining further confirmed these differences (Fig. 9B and C). These results highlight the role of surface topographies in modulating macrophage behaviour at both cytokine and morphological levels.

### 2.7 Minimally invasive glaucoma shunts with selected topographies

**Fig. 10:**
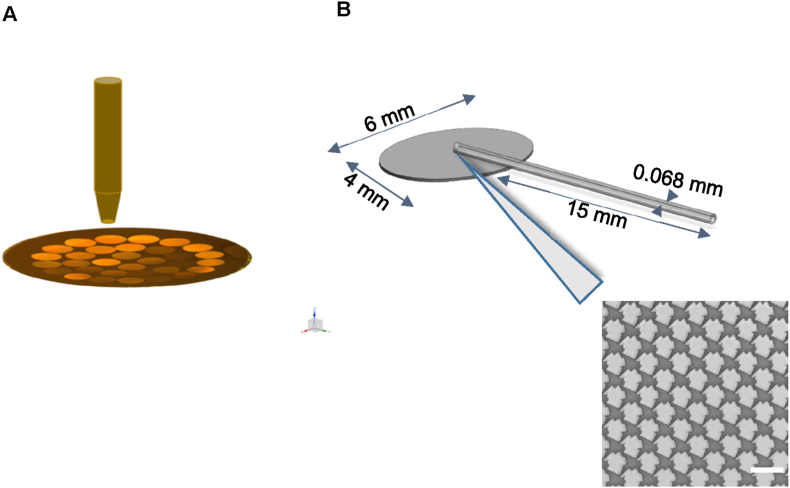
**Fabrication of topography modified micro shunts for *in vivo* analysis.** (A) Microshunts incorporating the three selected topographies for the *in vivo* rabbit model were fabricated, punched to the desired dimensions and attached to SIBS tubing via solvent welding. (B) Design parameters of the shunt device used for the *in vivo* studies showing the dimensions of the shunt tube along with the end plate imprinted with the topographies (T-1153) used for the rabbit study.

To assess the bioactivity of the three selected micro topographies *in vivo,* we manufactured minimally invasive glaucoma shunts with an endplate on which one of the three micro topographies is embossed. We also produced a shunt with a non-patterned endplate and another shunt in which we blocked the outflow of aqueous humor. The latter was a sham control for histological evaluation. Fabrication of flat and topography enhanced endplates was performed by hot embossing of 200 μm thick SIBS membranes. A metal bronze puncher was used to punch out oval membranes of 6 × 4mm (Fig. 10A) and the oval discs were attached to SIBS tubing with an inner lumen diameter of 68 ± 3 μm by solvent welding to obtain the design specifications chosen for the devices. The tubing was wired inside to ensure that the channels did not collapse during the solvent welding process. Once the endplates were attached, the fidelity of the micro topographies was tested (Fig. 10B) and the flow of the devices was tested (Video S1). All devices were then double packed for sterilization before performing the *in vivo* surgery. For each condition, fifteen implants were fabricated out of which seven devices were used for the implantation.

Supplementary data related to this article can be found online at https://doi.org/10.1016/j.bioactmat.2026.01.012

The following are the Supplementary data related to this article:

Multimedia component 2 Multimedia component 2

### 2.8 Intra ocular pressure drops after implantation

**Fig. 11:**
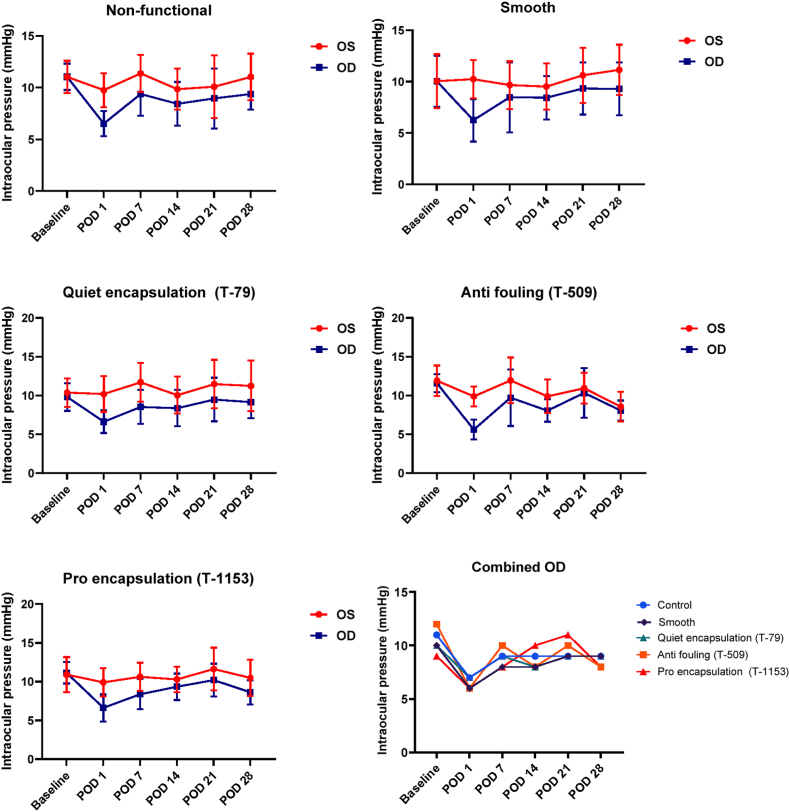
**Intraocular pressure (IOP) after implantation of SIBS microshunts.** Postoperative IOP measurements in eyes implanted with smooth (with or without flow) or topography-modified microshunts. All experimental eyes (OD) showed an initial IOP decrease, which remained lower than control eyes (OS) up to POD 14–28. No significant differences in IOP between groups at any time point were observed. Two-way ANOVA with Tukey post-hoc test (n = 7 per group).

IOP was monitored at regular intervals in both operated and unoperated eyes. In unoperated eyes, IOP remained stable at 10-11 mmHg throughout the experiment. In contrast, all operated eyes exhibited an immediate postoperative IOP drop to 4–6 mmHg on postoperative day (POD) 1, including the flow blocked control group, suggesting that surgical trauma and disruption of the blood aqueous barrier contributed to altered outflow dynamics. Over time, IOP gradually recovered in all experimental groups, though it remained slightly lower than in unoperated eyes. By POD28, all groups except the *anti-fouling* (T-509) group exhibited a residual 2 mmHg difference between operated and unoperated eyes (Fig. 11). However, due to inter animal variability, no statistically significant differences were detected between groups.

### 2.9 Histological evaluation of tissue formation at the implant interface

To assess the effect of surface micro topographies on tissue formation, histological analysis was performed at POD 28, using a quantitative scoring system (see Materials & Methods).

#### 2.9.1 Bleb height & collagen density

**Fig. 12:**
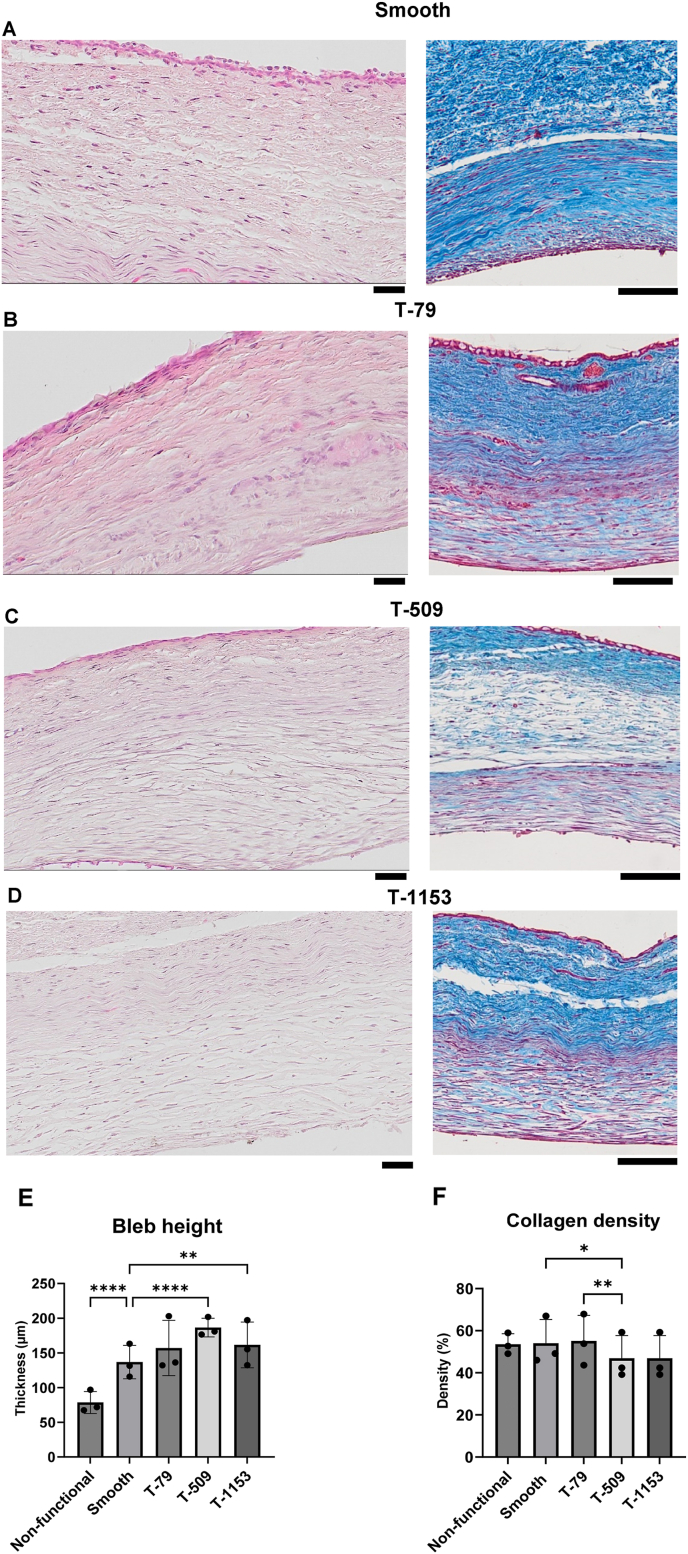
**Histological analysis of collagen and bleb height at tissue interface.** (A–D) H&E staining and Masson’s Trichrome–stained tissue sections from non-functional, smooth, T-79, T-509, and T-1153 groups at POD 28 (n = 7; three sections per animal) were used to assess bleb morphology and collagen organization. Smooth and T-79 exhibited tightly packed collagen, whereas T-509 and T-1153 showed larger blebs with loosely organized collagen (Scale bar: 50 μm). (E) Quantification of bleb height across groups, with each dot representing a measurement at different locations. (F) Collagen density within bleb tissue, with each dot representing a distinct location. Mean values from 21 experimental slides ± standard deviation is shown for each parameter. Statistical analysis was performed using one-way ANOVA with Tukey’s multiple comparisons test (n = 7 per group) (∗p < 0.05, ∗∗p < 0.01, ∗∗∗∗p < 0.0001).

H&E staining confirmed bleb height variations across groups (Fig. S4). T-509 exhibited the largest bleb height with the lowest collagen density, while T-79 and T-1153 had similar bleb height but differed in collagen organization, with T-1153 showing more resolved tissue structure (Fig. 12A–D). Masson's trichrome staining corroborated these findings. Tightly packed, thick collagen fibers were observed in T-79, while T-509 and T-1153 exhibited lower collagen density despite larger bleb height than the smooth group. These results further demonstrate the impact of surface micro topographies on bleb height and collagen organization.

Bleb height, measured at three locations along the endplate, reflects the tissue deposition from the implant interface to the conjunctival tissue. Non-functional implants formed a bleb height of 79 ± 16 μm, while implants with aqueous humor flow exhibited increased bleb height. The smooth endplate had a bleb height of 137 ± 14 μm. Surface topography significantly affected bleb height (Fig. 12E), with the *anti-fouling* topography (T-509) forming the larger bleb height. Interestingly, T-509 had the lowest collagen density, differing significantly from the smooth implant and *pro* *encapsulation* (T-1153) group (Fig. 12F). No difference in collagen density was observed between the smooth and non-functional implants, reinforcing that topography influences bleb architecture.

#### 2.9.2 Inflammatory response & neovascularization

**Fig. 13:**
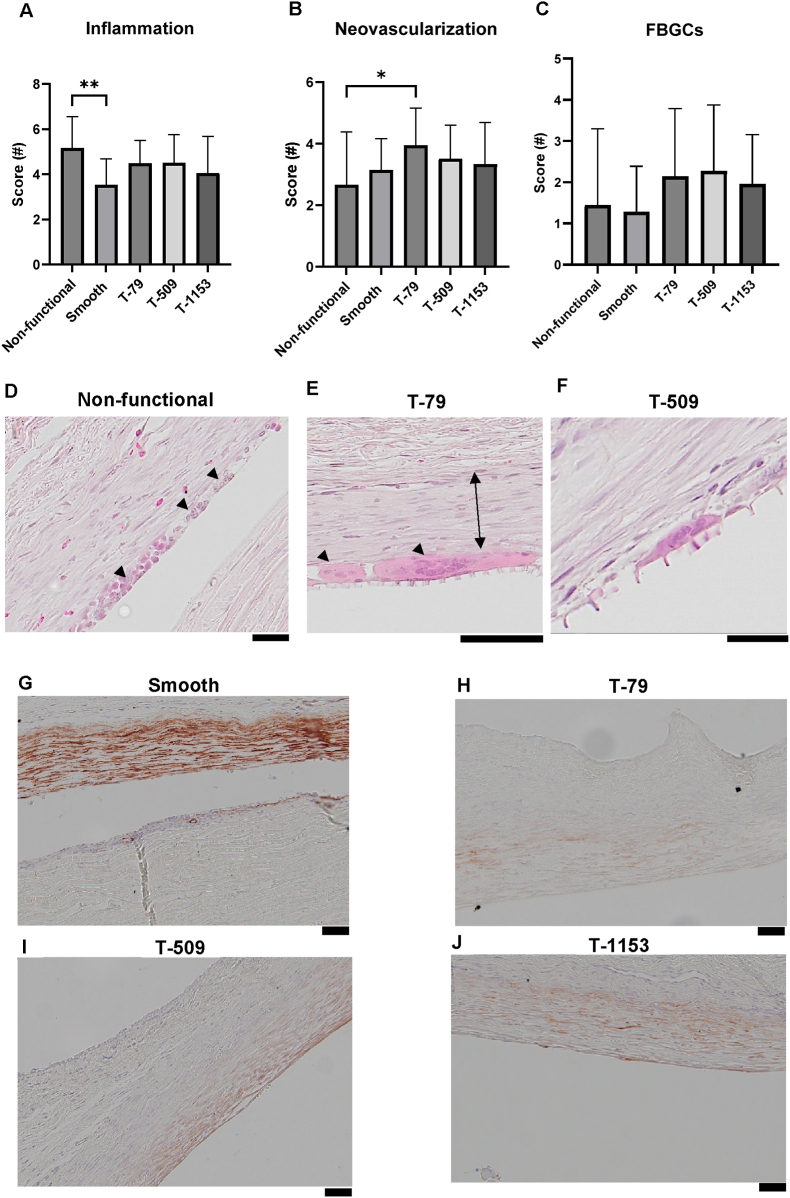
**Inflammation and α-SMA staining at the tissue interface.** Inflammatory response and α-SMA expression were assessed at POD 28 in non-functional, smooth, T-79, T-509, and T-1153 groups (n = 7; three sections per animal). (A–C) Bar plots show scores for inflammation, neovascularization, and foreign body giant cell (FBGC) formation, with scores ranging from 0 (“low”) to 6 (“high”). (D) Inflammation scores were highest in the nonfunctional implant group, consistent with the elevated number of macrophages observed (arrowheads). (E,F) FBGCs noted mainly in T-79 and T-509 between topographies and capsule (arrowheads). (G–J) α-SMA staining showed dense fibers at the interface for smooth implants compared with topography-modified surfaces (Scale bar: 50 μm).

Inflammatory scores did not differ significantly between groups, except for the smooth implants, which had lower inflammatory scores compared to the non-functional group (Fig. 13A). T-79 induced a significant increase in neovascularization compared to non-functional implants, likely due to aqueous humor flow into the bleb (Fig. 13B). Foreign body giant cells (FBGCs) were observed across groups, but differences were not statistically significant (Fig. 13C). Scoring revealed that non-functional implants had a heightened inflammatory response, with increased macrophages at the interface (Fig. 13D). FBGC presence varied between animals but was observed at the topography interfaces (Fig. 13E,F). As shown in the α-SMA–stained tissue sections (Fig. 13G–J), the smooth implant surface exhibited a dense, continuous band of α-SMA positive fibers at the interface, indicative of pronounced myofibroblast activation and a more contractile, fibrotic capsule. In contrast, all three topography groups displayed only mild and discontinuous α-SMA staining, with markedly fewer contractile fibers visible at the implant–tissue boundary. The reduction in α-SMA signal across these groups suggests that micro topographical features substantially attenuate myofibroblast differentiation and limit the formation of highly organized fibrotic tissue. Collectively, these observations support the conclusion that engineered micro topographies promote a more favorable, less fibrotic wound-healing response compared with smooth surfaces.

## 3 Discussion

This study explores the impact of micro topography on fibrotic response and bleb survival at the interface of GDDs. Unlike current GDDs that primarily rely on antifibrotic drugs to counter FBR, our approach focuses on modifying the surface architecture of the implant's endplate to direct fibrosis [40,41]. Our findings demonstrate that surface texture significantly influences cell behaviour, as confirmed by *in vitro* experiments. Moreover, bleb height varied between topographical groups, revealing key differences in capsule formation within the bleb and collagen density. Notably, smooth surfaces formed thinner capsules compared to topography textured surfaces, aligning with certain studies in rodent models where smooth silicone implants induced thinner but denser capsule [42]. However, other studies, such as Doloff et al., reported the opposite effect in a rabbit model, where smooth implants evoked thicker capsules compared to textured ones [7].

A critical distinction in our study is the consistent height of topographical features across all groups, with variations only in size and shape. This controlled design highlights the pivotal role of microtopography in modulating capsule architecture, reinforcing that surface modifications can strategically influence fibrosis at implant interfaces. This was addressed by selecting a well-established biocompatible polymer, SIBS which has been widely used in medical device fabrication, including stents and glaucoma shunt devices, and has demonstrated improved postoperative patency [25]. However, the hydrophobic nature of SIBS presents technical challenges for *in vitro* experiments. These challenges were addressed by modifying surface wettability through plasma treatment and by evaluating cell adhesion on micro topographies with and without plasma treatment as internal controls. This approach is supported by previous TopoChip screening studies using different polymers, which demonstrated that TDDs exert a dominant influence on immune cell attachment and mesenchymal stem cell proliferation, independent of the underlying polymer chemistry, including materials such as polycarbonate urethane and polystyrene [38,39].

In the context of GDD implantation, defining an ideal tissue response is crucial for long term success. A common misconception is that capsule thickness alone determines implantation success. However, in glaucoma surgery, the formation of a functional filtration bleb characterized by healthy, loosely packed tissue without excessive collagen deposition is essential to prevent bleb encapsulation and ensure sustained aqueous humor drainage [43]. Both overly thick and excessively thin capsules pose risks. A thick, fibrotic capsule may block filtration, leading to device failure, whereas a thin, underdeveloped bleb can result in over filtration, potentially causing hypotony and vision impairment [44]. These observations were substantiated by modelling the bleb in a study conducted by our collaborators [45]. Therefore, achieving a balanced fibrotic response that maintains controlled aqueous humor outflow is paramount for implant success. In this study, T-509 and T-1153 demonstrated the ability to induce a spongy, low density collagen matrix at the implant interface, facilitating filtration. While these findings are promising, long term studies (6–12 months) are necessary to evaluate bleb stability and functional longevity, which are critical before advancing toward clinical applications.

Our findings reveal that surface micro topographies on SIBS implants can modulate fibroblast and macrophage behavior, influencing tissue formation at the implant interface. *Anti-fouling* topographies (e.g., T-509) reduced fibroblast attachment and myofibroblast differentiation, resulting in lower collagen deposition while maintaining bleb height, suggesting a potential strategy to prevent fibrotic encapsulation, a major cause of glaucoma drainage device failure. *Pro* *encapsulation* surfaces (e.g., T-1153) promoted higher α-SMA expression and collagen deposition, highlighting designs that may exacerbate scarring and compromise implant patency. Additionally, micro topographies influenced macrophage attachment and cytokine secretion, with *quiet encapsulation* surfaces supporting anti-inflammatory profiles, indicating that immune modulation is achievable through surface design. To further strengthen the scientific impact of this work, future studies could incorporate mechanistic analyses such as gene expression profiling or macrophage–fibroblast co-culture experiments to elucidate the cellular and molecular pathways driving the topography dependent tissue responses observed here, as these approaches have previously revealed that macrophage behaviour, including cytokine secretion and migration, is strongly influenced by interactions with fibroblasts in the presence of biomaterials [46,47].

Although IOP differences were not significant within the 28-day study, histological markers such as bleb height, collagen density, and immune cell infiltration serve as early predictors of implant success. Clinically, these results suggest that carefully engineered surface micro topographies could improve post-surgical tissue remodeling, enhance long-term device patency, reduce fibrosis and inflammation, and ultimately improve patient outcomes following glaucoma surgery.

While this study provides key insights into tissue responses to micro topography, it does not fully elucidate the cellular interactions between infiltrating macrophages and fibroblasts at the implant interface. Cells sense the physical characteristics of an implant's surface (such as topography, stiffness, and spacing) primarily through integrin mediated focal adhesions, which link the extracellular matrix to the actin cytoskeleton and transduce mechanical signals [48,49]. These adhesions activate downstream signalling through focal adhesion kinase (FAK), Src, and associated proteins, which modulate cytoskeletal tension, stress fiber formation, and ultimately influence cell spreading, proliferation, and differentiation [50]. In addition, mechanotransduction converges on nuclear regulators such as YAP/TAZ when cells experience strong cytoskeletal tension often promoted by dense adhesion and rigid or appropriately patterned surfaces, YAP/TAZ translocate into the nucleus and drive transcriptional programs governing proliferation, survival, and extracellular matrix production [51]. Future investigations should focus on these dynamic cell interactions, potentially through gene expression profiling at different time points [52,53] or employing fibrosis disease models, knockout studies, and fluorescent reporter animals for real time tracking of immune activation and tissue remodeling [[54], [55], [56], [57]].

A notable achievement of our study was the successful integration of machine learning models to identify topographical design principles that modulate macrophage and fibroblast phenotypes, both central to the FBR [58,59]. By employing a high throughput combinatorial approach, we identified feature sizes and spacing that regulate fibroblast attachment and myofibroblast differentiation. Specifically, larger pillars with low spacing promoted fibroblast adhesion and α-SMA expression, while smaller features with greater spacing reduced cell attachment and contractility. These findings align with prior research demonstrating that topographical cues can direct fibroblast behaviour via mechanical signalling pathways, particularly Rho-ROCK signalling, which governs myofibroblast transdifferentiation [[60], [61], [62], [63]].

Beyond topographical design, this study adds a layer of complexity by examining the bleb tissue at the implant interface, which is influenced by aqueous humor derived growth factors. A rabbit study demonstrated that aqueous flow increases capsule thickness and α-SMA expression, highlighting its role in tissue remodeling [64]. Additionally, flow dynamics generate mechanical strain, further activating fibroblasts. This interplay between biochemical and mechanical cues is crucial in shaping the implant interface. We also observed that topographical designs differentially influence macrophage attachment and secretory phenotypes, aligning with previous findings [39]. Notably, elongated macrophages tend to polarize toward an anti-inflammatory phenotype underscoring the impact of surface topography on immune modulation. It aligns with existing research indicating that elongated macrophages tend to polarize towards an anti-inflammatory phenotype [4,65,66].

The complexity of tissue formation after implantation necessitates advanced *in vitro* test systems that replicate the implant environment. While coculture models have been used to study cell-cell interactions, traditional systems lack dynamic capabilities to track evolving FBR over time [14,67,68]. Nevertheless, traditional systems lack the dynamic capabilities required to assess the evolving parameters of the FBR, especially when considering how cell responses change over time. This limitation is evident in multiplex cytokine assays, where cytokine expression fluctuates at different time points. High throughput microfluidic platforms offer a promising solution by enabling real time investigation of immune and other key cell types involved in FBR. Leveraging these technologies can provide critical insights for designing biomaterials that optimize tissue response and improve implant outcomes [69,70]. Scaling up production of topography-enhanced glaucoma implants requires maintaining feature fidelity, consistent flow, and structural integrity during hot embossing, cutting, and solvent welding. Key constraints include preventing lumen collapse, ensuring uniform topographies, and preserving surface properties through sterilization, all of which demand robust quality control for reliable, high-throughput manufacturing.

Translating *in vitro* findings into *in vivo* setting remains a significant challenge, particularly in the context of GDD surgeries, where tissue formation is highly complex. Our study highlights that capsule formation alone is not necessarily detrimental to implant function; rather, the composition and architecture of the tissue play a crucial role in fostering a functional bleb. Notably, loosely packed collagen facilitates aqueous humor absorption, whereas dense tissue formation may contribute to implant failure. To further validate these insights, mechanistic assays are needed to elucidate cell-cell interactions within the implant interface. Additionally, assessing bleb patency over extended periods is essential to determine long term implant viability. Future studies should incorporate longitudinal *in vivo* assessments to evaluate the stability of the tissue response, ensuring optimal implant performance and longevity.

## 4 Conclusion

This study shows successful replication of micro topographies using biocompatible SIBS polymer with high fidelity that modulates phenotype both in human tenon fibroblasts and macrophages *in vitro*. It is shown that the design space of micro topographies plays a significant role in the modulation of cell phenotypes. This is further confirmed by the ability of these micro topographies to elicit distinct tissue responses *in vivo*, as evidenced by differences in bleb height and collagen density. This work advances our understanding of the interplay between biomaterial modified micro topographies and cell responses involved in fibrotic processes, paving the way for future developments in implant design and biocompatibility.

## 5 Materials and Methods

### 5.1 TopoChip fabrication

A detailed fabrication procedure of TopoChips is described elsewhere [37]. To summarize, 2176 unique micrometer range topographies were designed using an algorithm. Each topography was repeated on a TopoUnit of 280 × 280 μm^2^ and a mask was produced with this design. An additional mask was produced to allow the production of 20 μm high walls to separate the TopoUnits. Micro topographies are 10 μm in height. One TopoChip thus contained 2176 unique micro topographies repeated twice and contained four TopoUnits with unpatterned surfaces. The design was then inversely patterned onto a silicon wafer using directional reactive ion etching (DRIE), to create a master mold. Before production of the final SIBS TopoChips, two intermediate molds were prepared using PDMS and OrmoStamp®. The silica master mold was silanized using trichloro(1h,1h,2h,2h-perfluorooctyl)-silane (Merck), and PDMS (Dow corning) was used to create a positive mold which was in turn used to create a second negative mold which is called an Ormo mold using OrmoStamp® (micro resist technology Gmbh). This was finally used for hot embossing of SIBS polymer (Santen) which was melted at 160° for 15 min and then manual pressure was applied using a hot embossing tool (Specac) for 5 min. Later, 5-ton pressure across the wafer was applied and the temperature was reduced to a demolding temperature of 90 °C which lasts around 30 min during which the hot plates were recirculated with cool water.

The same process was applied for the large surface fabrication where each topography covered an area of 1.9 cm^2^ on a 100 mm wafer. For all the *in vitro* experiments SIBS topographies were plasma-treated using a plasma asher (Emitech K1050X) for 30 s using radio frequency (RF) power of 50 W at 10 sscm flow rate. Quality of micro topographies was assessed using a Sensofar profilometer and SEM imaging was performed on selected surfaces. The feature IDx numbers referred to in this manuscript represent the unique ID given to individual micro topographies during the design of the TopoChip.

### 5.2 Human tenon fibroblasts culture and screening

Three different screens were performed with SIBS Topochips. Each screen consisted of ten TopoChips and each Topochip has two replicates of the same topography. Each screen was performed on the same day. For the first two screens, human tenon fibroblasts (HTFs) used in this study were isolated from primary human tenon tissue samples from glaucoma patients at the University Eye Clinic in Maastricht with permission from the local ethical committee (permission number 2019-0983). All the cells used in this study were from a single donor. During the study, human tenon fibroblasts were grown in a complete medium composed of advanced DMEM (Gibco) medium supplemented with 10 % FBS (Sigma) and Penicillin/Streptomycin (100 U/ml; Gibco) along with 0.2 mM of L-Glutamine (Gibco). Cells were grown in a humidified incubator at 37 °C with 5 % CO_2_ and the medium was changed every 2–3 days until 80 % confluency was reached. Cells were seeded at 10,000/cm^2^ at passage 4–5 unless mentioned otherwise. For the first screen, human tenon fibroblasts were seeded onto TopoChips placed in a six-well plate, and custom-made 3D printed inserts were used to place the TopoChips in place to avoid the movement of TopoChips after the addition of medium. Topochips were stimulated with 10 ng/ml TGF-β1(Peprotech) in the medium for 48 h and stained for αSMA as described below.

For the second screen, human tenon fibroblasts were seeded onto the TopoChips in a complete medium and incubated overnight. Then the Topochips were washed with PBS and serum-free medium was added. After 24 h, the medium was changed to a complete medium supplemented with 10 μM EdU (Invitrogen) grown for 48 h and stained for EdU as described below.

### 5.3 Monocyte isolation and screening

Peripheral blood mononuclear cells (PBMCs) were isolated from buffy coats of healthy donors (Sanquin) using density gradient centrifugation with Lymphoprep™ (Stem cell technologies) and subsequent magnetic cell separation using CD14 microbeads (Miltenyi Biotech). Purified monocytes from two different donors were separately resuspended in RPMI-1640 medium (Gibco) supplemented with 10 % FBS and Penicillin/Streptomycin (Gibco; 100 U/ml) and 100 ng/ml GM-CSF (Sigma) and seeded onto TopoChips with a seeding density of 600,000/cm^2^. The Topochips were incubated for 10 days, and the medium containing 100 ng/ml of GM-CSF was refreshed on day 3 and day 7. We repeated the same procedure for validation experiments.

### 5.4 Immunofluorescent staining and microscopy

After cell culture, TopoChips or large topographical surfaces were washed three times with sterile phosphate-buffered saline (PBS; Gibco) and fixed with 3.7 % (v/v) paraformaldehyde (Sigma- Aldrich) for 15 min at room temperature. Next, the cells were permeabilized with 0.5 % Triton X-100 (Merck) dissolved in PBS for 10 min and blocked with 3 % (w/v) bovine serum albumin (Merck) in PBS for 30 min and then with 5 % (v/v) goat serum (Fisher Scientific) dissolved in PBS for 30 min. Next, cells were incubated with a primary antibody using mouse monoclonal anti α-smooth muscle actin diluted in PBS (1:600; Sigma: A5228) overnight at 4 °C. The next day, the cells were washed 3 times with PBS and then incubated with a secondary antibody goat anti-mouse Alexa Flour 488 diluted in PBS (1:500; Molecular probes: A21121) and incubated in the dark for 1 h at room temperature. For the α-SMA screen, cells were washed three times with PBS and stained with phalloidin-TRITC diluted in PBS (1:200, ThermoFisher) for 45 min and washed 3 times again with PBS and stained with 4′,6-Diamidino-2-Phenylindole (DAPI) (Sigma Aldrich; 1:500) for 15 min. For the macrophage screen, the cell membrane was stained using CellMask™ orange (1:1000; ThermoFisher) for 10 min at room temperature. Afterward, cells were washed with PBS 3 times and stained with DAPI (Sigma Aldrich; 1:500) for 15 min. After staining, all the surfaces were washed three times with PBS after which they were mounted onto a glass slide using Mowiol (Sigma). All surfaces were imaged using a Nikon Eclipse Ti2 high-content imaging microscope.

### 5.5 Image analysis

All TopoChips were imaged as a tile scan of the whole 2 × 2 cm^2^ TopoChip surface, and we used a MATLAB script to crop the images down to individual TopoUnits, and all images were then stitched to a 66 × 66 TopoUnit image. Cell staining and distribution were checked for all chips. All the cropped images were then fed into a custom-designed CellProfiler pipeline, i.e. each screen had one pipeline. We used the DAPI signal to identify the nucleus as a primary object and then used phalloidin (HTFs) or CellMask Orange (macrophage) to define the cell as a secondary object. For the α-SMA screen, we quantified the integrated intensity α-SMA in the secondary object as readout, and for the HTF and macrophage attachment screens, we calculated the number of cells attached on each topography. Then, ranking was performed using the readout from each screen and these ranked topographies were further used to perform the classification models. All the corresponding pipelines are provided on our lab's git hub page (https://github.com/cbite/Glaucoma_screening.git). The reproducibility of readouts from each TopoChip screen integrated intensity of α-SMA, cell number of HTFs, and cell number of macrophages between the duplicates was determined by performing Kolmogorov-Smirnov (KS) and Anderson-Darling (AD) tests. All the topographies with low P-values of less than 0.05 were eliminated from rank plots. The significance of the corresponding readout values of high α-SMA and low α-SMA was evaluated with Kolmogorov-Smirnov (KS) test.

### 5.6 Classification of readouts with topographical design space and cell morphological parameters

To find TDDs that correlate to HTF cell number, α- SMA expression, and macrophage cell attachment, the top 300 highest and lowest ranking topographies were taken from the individual screens. The topographies were used as input for the machine learning model where the data was split into 25 % test data and 75 % trained data. The XGB classifier is used which is an eXtreme Gradient Boost algorithm based on gradient boosted decision tree algorithm in Python using Jupyter Notebook. The accuracy of this trained model was tested, and the receiver operating characteristic (ROC) curve was plotted which gives the measure of the classification. We also plotted the top TDDs that influenced the classification based on its importance which is given a weightage. To understand the model, the SHAP module was used to calculate the values of each TDDs which contributed to the model. These values can be positive or negative based on their impact on the model, and these were plotted based on the features of importance.

### 5.7 Multiplex ELISA

Purified monocytes from two different healthy human donors were separately resuspended in RPMI-1640 medium (Gibco) supplemented with 10 % FBS and Penicillin/Streptomycin (Gibco; 100 U/ml) and 100 ng/ml GM-CSF (Sigma) and seeded onto 32 selected topographies in duplicates with a seeding density of 600,000/cm^2^. Cells were incubated for 10 days, and the medium was refreshed on day 3 and day 7. After day 3 and day 6, 500 μl of supernatant from each condition was collected and stored at −80 °C until further analysis. Protein quantification was performed using the 13-plex LEGENDplex Human Macrophage/Microglia panel (BioLegend), for detecting IL-12p70, TNF-α, IL-6, IL-4, IL-10, IL-1β, Arginase, CCL17 (TARC), IL-1RA, IL-12p40, IL-23, IFN-γ and CXCL10 (IP-10), or the Human Free Active/Total TGF-β1 Panel (BioLegend), for detecting total TGF-β1 after release of complexed (inactive) TGF-β1 by acid treatment. Of these, TNF-α, IL-6, IL-10, IL-1β, Arginase, IL-1RA and total TGF-β1 were detectable.

The assay was performed according to the manufacturer's instructions with the following modifications. 5 μl of sample or standard was mixed in V-bottom polypropylene 96-well plates with 5 μl of assay buffer, beads, and detection antibodies and incubated at room temperature, shaking at 800 rpm. After 2 h, 5 μl of streptavidin-phycoerythrin (SA-PE) dye was added to each well, and plates were incubated for 30 min. Wells were then washed by adding 150 μl wash buffer, centrifuging at 1000×*g*, and removing the supernatant. Beads were resuspended in 80 μl wash buffer and fluorescence intensity was measured on a BD FACSCanto II flow cytometer. Data was analyzed using the BioLegend LEGENDplex™ Data Analysis Software Suite for gating, standard curve fitting, and determination of analyte concentrations, following the manufacturer's instructions.

### 5.8 In vivo experiments

Animal procedures were conducted according to the Association for Research in Vision and Ophthalmology (ARVO) Statement for the Use of Animals in Ophthalmic and Visual Research, the Animal Research: Reporting of *In Vivo* Experiments (ARRIVE) 2.0 guidelines [71], and the Guidelines of the Central Laboratory Animal Facility of Maastricht University. All protocols were approved by the Central Authority for Scientific Procedures on Animals (CCD, Den Haag, NL) and were in accordance with the European Guidelines (2010/63/EU) (Approved Dutch license number: AVD1070020209684). New Zealand White (NZW) rabbits (2.0–2.5 kg, males and females) (Envigo (Horst, NL and Bicester, UK) and Charles River (Ecully, FR)) were group housed (maximum 7 animals per cage, males and females separated) and maintained under controlled conditions of temperature and humidity on a 12h:12h light–dark cycle. The rabbits had *ad libitum* access to water and dried chow. All animals received a two-week acclimatization period before the start of the experiments. On postoperative day (POD) 27, the animals were injected twice (morning and late afternoon) with 30 mg/kg bromodeoxyuridine (BrdU, Abcam, Cambridge, UK) intravenously. At the end of the experiment, the rabbits were killed with an overdose of pentobarbital sodium, 200 mg/kg (Euthasol 20, Produlab Pharma B.V., NL), intravenously injected.

In a normal rabbit eye, the intra ocular pressure (IOP) is 12 ± 2 mmHg. During the experiment, an IOP lowering of at least 3 mmHg was expected, with a probability (α) of 5 % and a power (β) of 80 %. Prior data indicates that the difference in the response of matched pairs (experimental eye and the contralateral eye as the control eye) is normally distributed with a standard deviation of 2 mmHg. If the difference in the mean response of matched pairs is 3 mmHg, 7 subjects per group are needed (which includes a dropout of 10 % due to unforeseen circumstances). Thirty-five normotensive New Zealand White rabbits were ordered (21 females, 14 males). A randomized complete block design was used to allocate animals to the 5 groups (Table 1). To prevent bias, researchers were blinded until all data was collected and analyzed.

**Table 1:** Number of animals and their gender per group.

Implant	Females	Males
Smooth	5	2
Non-functioning (smooth)	4	3
T-79 (*Quiet encapsulation)*	4	3
T-1153 (*Pro encapsulation)*	4	3
T-509 (*Anti-fouling)*	4	3

### 5.9 Implantation of the shunts

The surgical procedure was like previous experiments [72]. In short, the right eye was always implanted, and all surgeries were performed by a single surgeon assisted by a second surgeon. Local analgesia was provided in the form of 0.4 % oxybuprocaine hydrochloride drops (MINIMS, Bausch & Lomb Pharma, Brussels, BE). A fornix-based conjunctival flap was dissected with Westcott tenotomy scissors, after which a 1 mm wide and 1 mm deep scleral pocket was formed with a side-port knife (ClearCut®, Alcon, Geneva, CH), 2.5 mm–3 mm posterior to the limbus. Implants were rinsed with sterile buffered 0.9 % saline solution and flow of the implants was confirmed by inserting the tube into a 23G needle to rinse the inner lumen of the implants. The endplate was placed under the conjunctival flap. Through the scleral pocket, a needle tract was made with a 25G needle into the anterior chamber. The tube was inserted through the pocket and needle tract into the anterior chamber. Hereafter, the conjunctiva and Tenon's capsule were sutured closed with a Vicryl 9-0 suture (Ethicon LLC, San Lorenzo, PR, USA). Animals were treated with 1 % chloramphenicol ointment twice daily, for 5 days and received a subcutaneous injection of buprenorphine (0.05 mg/kg Bupaq Multidose, Richter Pharma AG, Wels, AT) up until POD 1 three times daily, if needed analgesia was extended.

### 5.10 IOP measurements

From all rabbits, the IOP was measured three times from both eyes using the iCare TonoVet (iCare Finland Oy, Vantaa, FI) (in a dog/cat setting). To this end, the rabbits were sedated using 0.40 mg/kg medetomidine (Sedator, A.S.T. Farma B.V., Oudewater, NL) intramuscularly (IM). The rabbits were placed in a restrainer, and photographs were made from the experimental eye using a photo camera (Canon EOS 4000D, Canon, Tokio, JP). The eye was inspected using a slit lamp bio-microscope (Haag-Streit BI 900, Haag-Streit, Köniz, CH). To check corneal thickness and anterior chamber depth, anterior chamber optical coherence tomography (AC-OCT) was performed, using a SL-OCT module (Heidelberg Engineering, Heidelberg, DE) mounted on a slit lamp bio-microscope (Haag-Streit BD 900, Haag-Streit, Köniz, CH). Two days before the surgery (POD -2 and POD -1) baseline checks were performed. Other check-ups took place at POD 1, 7, 14, 21, and 28.

### 5.11 Histology and light microscopy

*Postmortem*, the eyes were dissected and fixed in 4 % paraformaldehyde (PFA) for 2 days (PFA was refreshed once a day). After fixation, the eyes were dehydrated and embedded in paraffin blocks. Sections were cut with a thickness of 5 μm and were stained with Hematoxylin & Eosin (H&E), Masson's Trichrome (Sigma-Aldrich, Merck Life Science NV, Amsterdam, NL), and mouse-anti-alpha-smooth muscle actin (α-SMA) (ThermoFisher Scientific, US, MA5-11547, 1:500). In the case of anti α-SMA, slides were blocked using 3 % hydrogen peroxidase and 10 % donkey serum (Abcam, UK, ab7475). A secondary antibody (donkey-anti-mouse, Jackson immune research, UK, 715-065-151, 1:2000 and donkey-anti-sheep (LSBio, US, LS-C61150, 1:3000)) with a biotin label was used to detect the primary antibody. Lastly, an ABC kit (Vector laboratories, US, PK6100) was used to enhance the biotin label for staining and Novared (Vector laboratories, US, SK-4800) was used to stain the slides. BrdU staining was not performed as the staining was too weak to perform analysis. Histological slides were assessed and semi-qualitatively scored by two blinded observers, after which photographs were taken using a confocal scanning microscope (BX-51, Olympus, JP), as performed previously [72]. To study the immune response and neo vascularization, scoring was performed across the biomaterial interface facing the conjunctival side with three tissue sections from the anterior, central, and posterior side of the eye's cross-section taken from each animal per group. Similarly, capsule thickness and collagen density were measured using ImageJ. All the measurements between the observer per tissue section were averaged and the average of these averages were plotted. A one-way ANOVA with Tukey post hoc comparisons was performed with the *in vivo* data with all the groups (n = 7) using Prism (GraphPad Software, San Deigo, CA).

### 5.12 α-SMA staining

Animals were euthanized using 200 mg/kg pentobarbital sodium (Euthasol 20, Produlab Pharma B.V., NL). After euthanasia, the eyes were dissected and fixed in 4 % paraformaldehyde (PFA) for 2 days (PFA was refreshed once a day), and after fixation, eyes were dehydrated and embedded in paraffin blocks. Sections were cut with a thickness of 4 μm and were stained with Hematoxylin & Eosin (H&E), mouse-anti-alpha-smooth muscle actin (*α*-SMA) (ThermoFisher scientific, US, MA5-11547, 1:500). In the case of anti-*α*-SMA slides were blocked using 3 % hydrogen peroxidase and 10 % donkey serum (Abcam, UK, ab7475). A secondary antibody (donkey-anti-mouse, Jackson immunoresearch, UK, 715-065-151, 1:2000 with a biotin label was used to detect the primary antibody. Lastly, an ABC kit (Vector laboratories, US, PK6100) was used to enhance the biotin label for staining and Novared (Vector laboratories, US, SK-4800) was used to stain the slides.

### 5.13 Statistical analysis

All experimental data in this study with statistical analysis were performed with mean ± standard deviation of the means. T-test and analysis of variance (ANOVA) were used to compare the two groups or more than two groups. GraphPad Prism (version 10.2) was used to calculate the significance level of the data (∗∗∗*p* < 0.0001, ∗∗∗*p* < 0.001, ∗∗*p* < 0.01, ∗*p* < 0.05).

## CRediT authorship contribution statement

**Phani Krishna Sudarsanam:** Writing – review & editing, Writing – original draft, Visualization, Validation, Software, Methodology, Investigation, Formal analysis, Data curation, Conceptualization. **Ralph J.S. van Mechelen:** Validation, Methodology, Investigation, Formal analysis. **Tim J.M. Kuijpers:** Visualization, Software, Investigation, Formal analysis, Data curation. **Christian J.F. Bertens:** Visualization, Validation, Methodology, Data curation. **Els Alsema:** Methodology, Investigation. **Juul Verbakel:** Visualization, Investigation. **Nick R.M. Beijer:** Writing – review & editing, Supervision. **Henny J.M. Beckers:** Writing – review & editing, Supervision, Resources, Project administration, Funding acquisition, Conceptualization. **Jan de Boer:** Writing – review & editing, Writing – original draft, Supervision, Resources, Project administration, Methodology, Funding acquisition, Conceptualization.

## Ethics approval and consent to participate

Animal procedures were conducted according to the Association for Research in Vision and Ophthalmology (ARVO) Statement for the Use of Animals in Ophthalmic and Visual Research, the Animal Research: Reporting of *In Vivo* Experiments (ARRIVE) 2.0 guidelines [65], and the Guidelines of the Central Laboratory Animal Facility of Maastricht University. All protocols were approved by the Central Authority for Scientific Procedures on Animals (CCD, Den Haag, NL) and were in accordance with the European Guidelines (2010/63/EU) (Approved Dutch license number: AVD1070020209684).

## Declaration of competing interest

The authors declare the following financial interests/personal relationships which may be considered as potential competing interests: Jan de Boer reports financial support was provided by Chemelot Institute for Science and Technology (InSciTe) under Grant BM3.03 SEAMS. If there are other authors, they declare that they have no known competing financial interests or personal relationships that could have appeared to influence the work reported in this paper.
