# Supplementary material for: Immunomodulatory topographies regulate myofibroblast differentiation and influence fibrous encapsulation of glaucoma drainage devices
Source: Bioact Mater. 2026 Feb 2;60:492–509. doi: 10.1016/j.bioactmat.2026.01.012 (PMC12887268; doi:10.1016/j.bioactmat.2026.01.012)
Supplement: Multimedia component 1 [file mmc1.docx]

**Supporting Information**


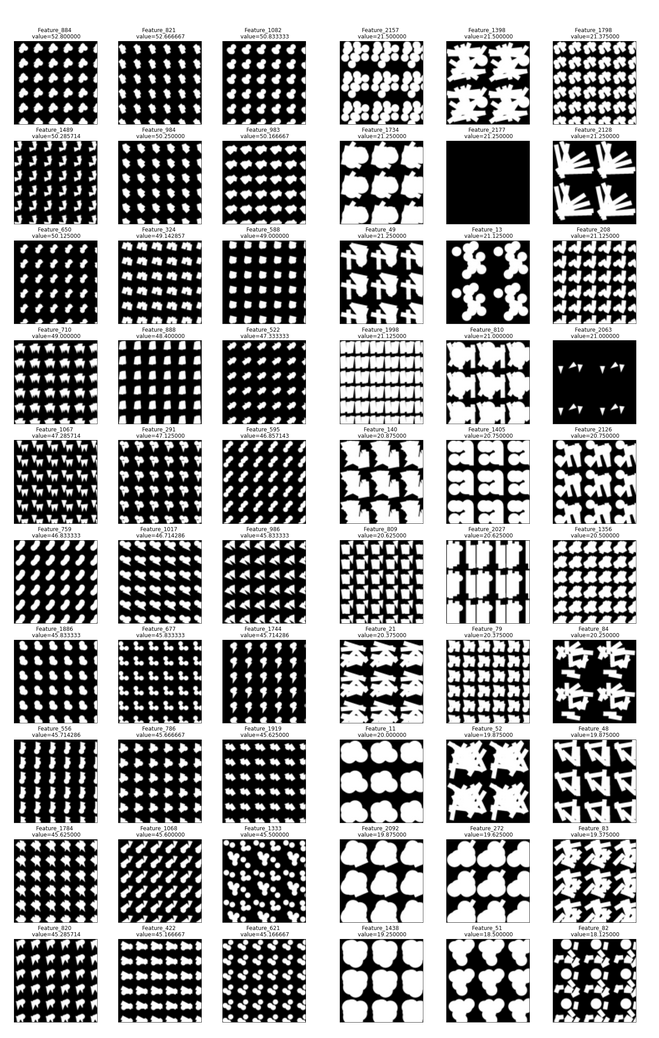


**Figure S1. Macrophage cell count on SIBS TopoChips not treated with oxygen plasma.** Images of high and low macrophage count topographies for top and bottom 30 topographies show the differences in the size of the features and the distance between the features, similar to what is observed in plasma treated TopoChips.

**Low α-SMA**

**High α-SMA**


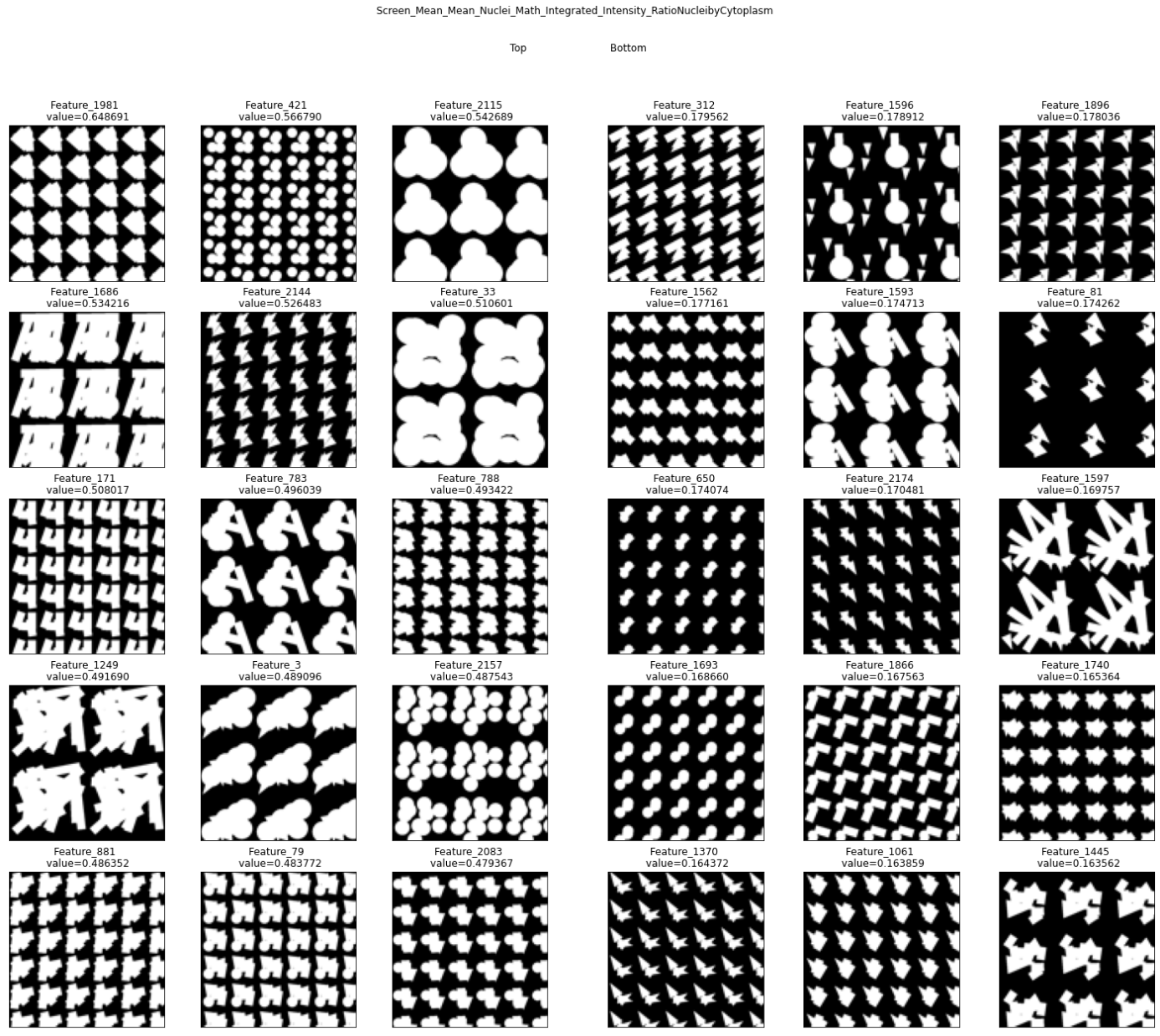


**Figure S2. Binary images of high and low α-SMA hits.** Design images of high α-SMA and low α-SMA topographies show the differences in the size of the features and the distance between the features.

Pro encapsulation

Quiet encapsulation

Anti fouling

**A**

**B**

**Figure S3. Validation experiments for the three chosen conditions from the TopoChip screens.** A) Scatter plot showing macrophage cell number and fibroblast cell number from chosen 32 different topographies. T-509 (circled in green) for *anti-fouling*, T-79 (circled in blue) for *quiet encapsulation* were chosen for animal studies. B) Scatter plot showing macrophage cell number and mean α-SMA intensity from the validation study performed with chosen topographies. T-1153 (circled in red) was chosen for *pro encapsulation* for animal study. Flat surface represented by .


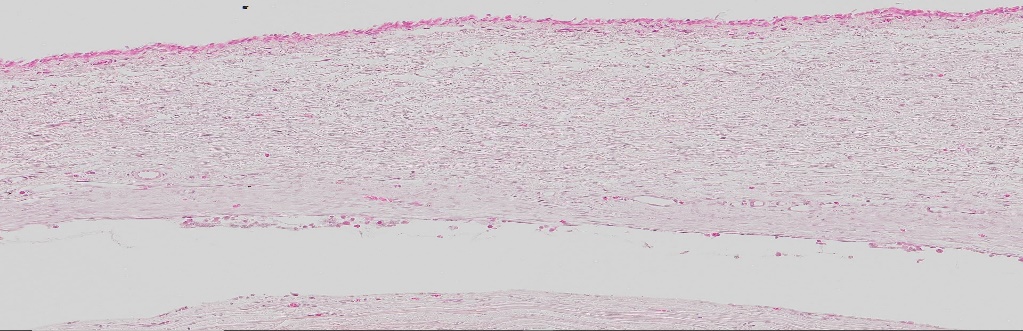


Non-functional


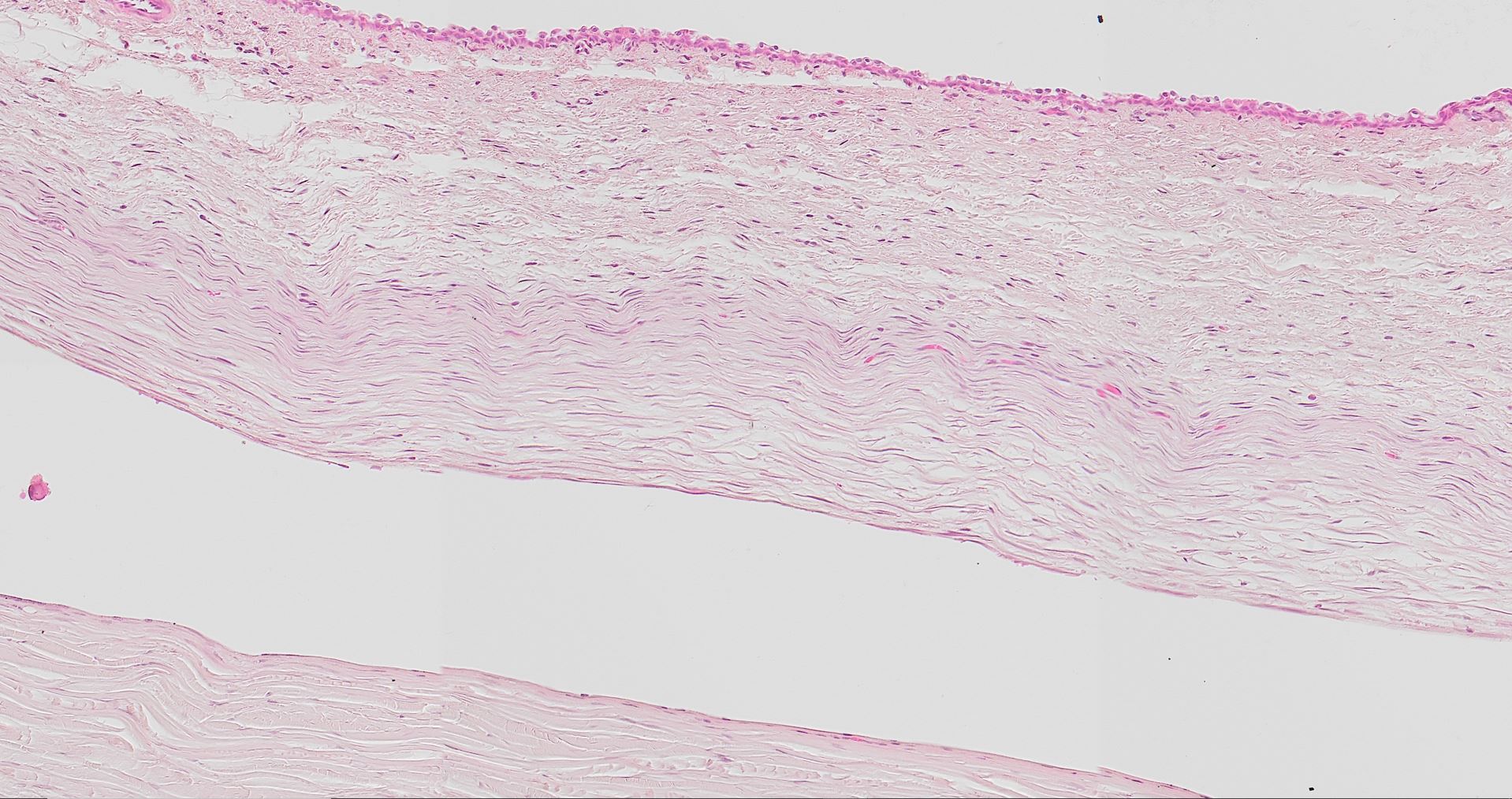

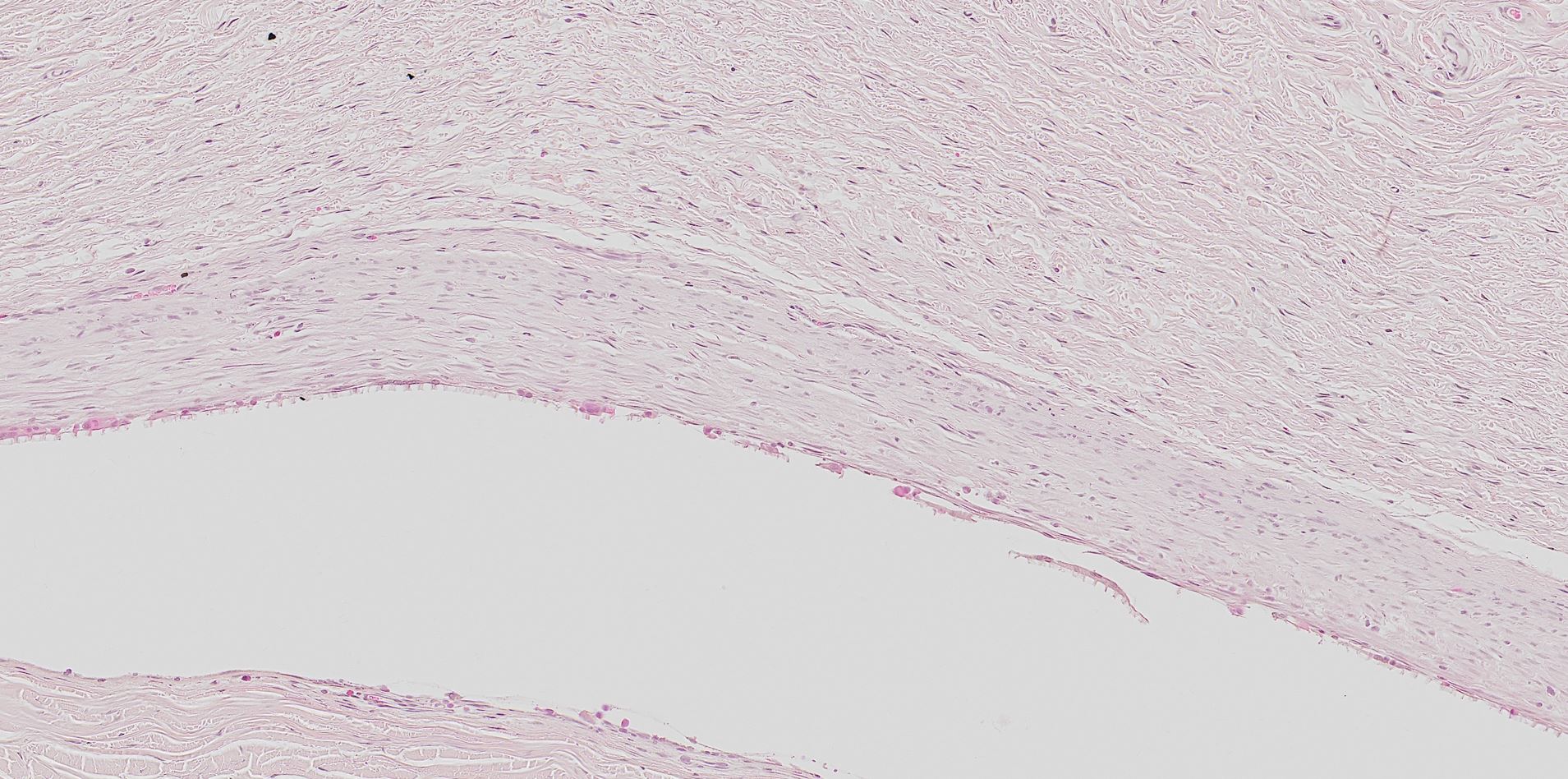

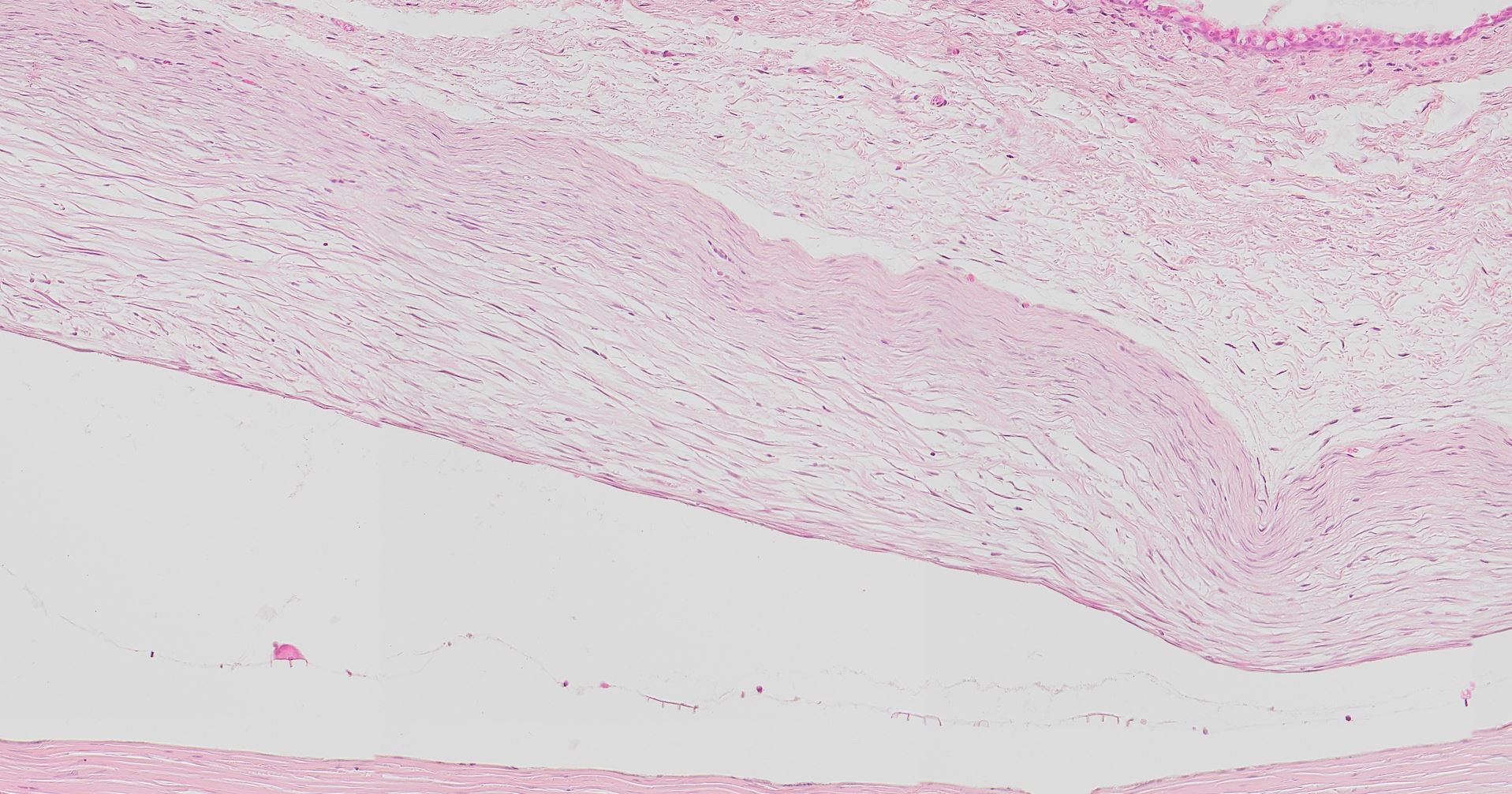

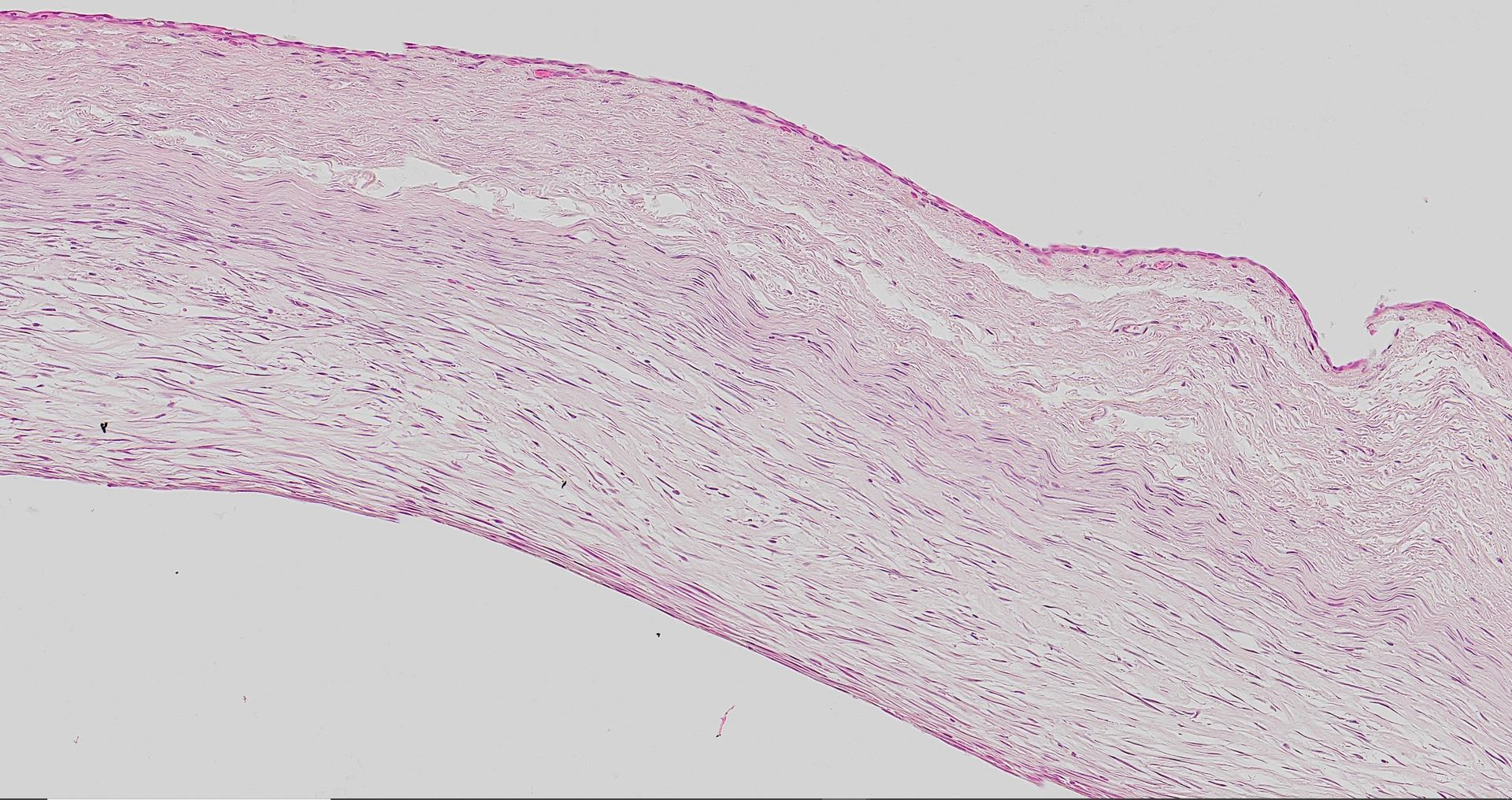


Bleb

Endplate

Sclera

Smooth

T-79

T-509

T-1153

**Figure S4. Histological images of tissue sections.** H&E-stained tissue sections scanned confirming the quantified observations. Stained sections of all the groups with non-functioning, smooth, T-79, T-509, T1153 show the differences in inflammation, capsule thickness between the groups at the implant interface. Scale bar: 50 µm.
